# Developing an experimental necrotic enteritis model in turkeys - the impact of *Clostridium perfringens*, *Eimeria meleagrimitis* and host age on frequency of severe intestinal lesions

**DOI:** 10.1186/s12917-020-2270-5

**Published:** 2020-02-18

**Authors:** Simon P. Hardy, Sylvie L. Benestad, Inger Sofie Hamnes, Torfinn Moldal, Bruce David, John R. Barta, Jean-Michel Reperant, Magne Kaldhusdal

**Affiliations:** 1 University of Brighton, Lewes Road, Brighton, BN2 4GJ UK; 2 Norwegian Veterinary Institute, P.O.B. 750 Sentrum, 0106 Oslo, Norway; 3 Nortura SA, Sloraveien 60, 1878 Haerland, Norway; 4 University of Guelph, 50 Stone Road East, Guelph, ON N1G 2W1 Canada; 5 Anses, Laboratory of Ploufragan-Plouzané-Niort, 22440 Ploufragan, France

**Keywords:** Necrotic enteritis, Challenge model, Turkeys, Macroscopic lesions, Scoring system, *Clostridium perfringens*, *Eimeria meleagrimitis*, Age, Refinement

## Abstract

**Background:**

Necrotic enteritis is a significant problem to the poultry industry globally and, in Norway up to 30% of Norwegian turkey grow-outs can be affected. However, despite an awareness that differences exist between necrotic enteritis in chickens and turkeys, little information exists concerning the pathogenesis, immunity, microbiota or experimental reproduction of necrotic enteritis in turkeys. In particular, it is important to determine the appearance of the gross lesions, the age dependency of the disease and the role of netB toxin of *Clostridium perfringens*. To this end, we report our findings in developing an in vivo experimental model of necrotic enteritis in turkeys.

**Results:**

A four tier (0–3) scoring system with clearly defined degrees of severity of macroscopic intestinal lesions was developed, based on 2312 photographic images of opened intestines from 810 B.U.T. 10 or B.U.T. Premium turkeys examined in nine experiments. Loss of macroscopically recognizable villi in the anterior small intestine was established as the defining lesion qualifying for a score 3 (severe intestinal lesions). The developed scoring system was used to identify important factors in promoting high frequencies of turkeys with severe lesions: a combined *Eimeria meleagrimitis* and *Clostridium perfringens* challenge, challenge at five rather than 3 weeks of age, the use of an *Eimeria meleagrimitis* dose level of at least 5000 oocysts per bird and finally, examination of the intestines of 5-week-old turkeys at 125 to 145 h after *Eimeria meleagrimitis* inoculation. Numbers of oocysts excreted were not influenced by *Clostridium perfringens* inoculation or turkey age. Among three different lesion score outcomes tested, frequency of severe lesions proved superior in discriminating between impact of four combinations of *Clostridium perfringens* inoculation and turkey age at challenge.

**Conclusions:**

This study provides details for the successful establishment of an in vivo model of necrotic enteritis in turkeys.

## Background

*Clostridium perfringens*-associated necrotic enteritis is an important disease in intensive poultry farming. The causative agent [1, 2], pathogenesis [3, 4], role of host immunity [5] and intestinal microbiota [6, 7], epidemiology [8, 9] and experimental reproduction [10] of this disease in broiler chickens have been extensively investigated. Necrotic enteritis occurs in turkeys and *Clostridium perfringens* has been implicated in the aetiology [11–15], but little information exists concerning the pathogenesis [16], immunity, microbiota or experimental reproduction of turkey necrotic enteritis.

Experimentally-induced necrotic enteritis in chickens has been critical in the study of the disease [17], and the lack of an experimental model of turkey necrotic enteritis represents an obstacle to improve the understanding of the disease in that host. Combined administration of coccidia and *Clostridium perfringens* is considered an efficient way of inducing experimental necrotic enteritis in chickens [10], and similarly the combination may be useful in turkeys, since data from commercial turkey flocks suggest a predisposing role of coccidia in turkey necrotic enteritis [13, 14]. *Eimeria meleagrimitis* is a pathogenic coccidium capable of inducing lesions mainly in the duodenum and jejunum [18]. In our experience (unpublished data) and according to other workers [11, 15], the anterior small intestine is most frequently and most severely affected by necrotic enteritis in turkeys thereby implicating a predisposing role for *Eimeria meleagrimitis* in necrotic enteritis of turkeys.

Scoring systems for macroscopic intestinal lesions are valuable when assessing impact of influencing factors on disease outcome in necrotic enteritis models [10]. Ideally such scoring systems distinguish clearly between degrees of lesion severity, making it possible to score consistently within and between experiments. If possible, the scoring system should also include necrotic enteritis specific scores. Anterior small intestinal mucosal ulcers/depressions and pseudomembranes are considered necrotic enteritis specific macroscopic lesions in chickens [19–21]. These lesions are usually easy to identify and distinguish from macroscopic lesions caused by coccidia inducing lesions in the anterior small intestine, but the frequency of such lesions may be variable in experimental chicken models. Most chicken models are therefore based on scoring systems involving additional and less specific macroscopic criteria. Current knowledge about how to differentiate between macroscopic intestinal lesions in turkeys caused by *Eimeria meleagrimitis* and *Clostridium perfringens* is limited.

This work describes a new scoring system with defined degrees of severity for macroscopic intestinal lesions in *Eimeria meleagrimitis*- and *Clostridium perfringens*-inoculated turkeys, and investigates the impact of *Clostridium perfringens* inoculation and other factors on the frequency of severe lesions in turkeys inoculated with *Eimeria meleagrimitis*, as a contribution to the development of a necrotic enteritis challenge model in turkeys.

## Results

### A macroscopic scoring system for intestinal lesions

**Fig. 1:**
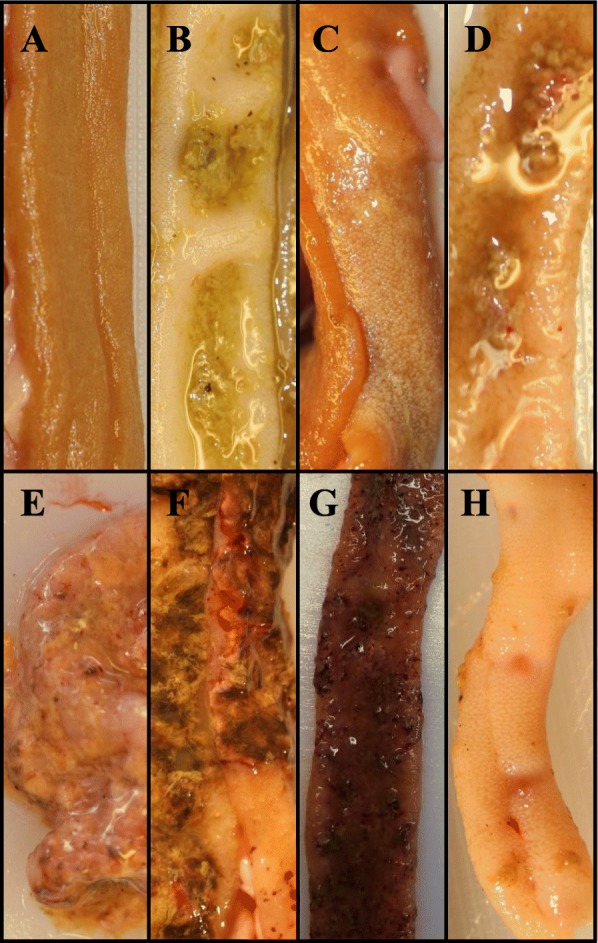
Photos of small intestinal mucosa from turkeys. A: Score 0 (normal). B: Score 1 (mild changes: pale mucosa and increased amount of watery intestinal contents). C: Score 2 (moderate changes): swollen, pale villi. D-G: Score 3 (various sub-types of severe changes). H: Score 3 (mucosal depressions suggesting healing multifocal necrotic enteritis). Photos A-F and H: Simon P Hardy. Photo G: Magne Kaldhusdal

A macroscopic scoring system with four categories (0–3) was developed. The criteria for each score were as follows:

0 Normally coloured intestinal mucosa. Non-dilated intestine that everts rapidly and completely after being opened longitudinally. Normal intestinal contents. Transparent and inconspicuous villi (Fig. 1a).
1 Defining characteristic: Pale anterior (entirely or partly from the gizzard to Meckel’s diverticulum) small intestinal mucosa. Intestine may evert incompletely after longitudinal opening. Villi are not conspicuous but may be slightly opaque or swollen, or the intestinal mucosa may be partly hyperaemic with reddish villi. There is usually increased amount of non-viscous, transparent fluid in intestinal contents (Fig. 1b).
2 Recognizable villi are present. The villi are clearly opaque or swollen and/or the mucosa shows moderate swelling, oedema or petechial haemorrhages. There may be blood clots and/or turbid (often mucoid) non-adherent materials in the intestinal lumen. Changes found in intestines with score 1 may also be present (Fig. 1c).
3 Defining characteristic: Loss of recognizable villi (regenerating villi may be present beneath a sloughing necrotic mucosa). Severely swollen and marbled mucosa with glossy surface and/or mucosa covered diffusely or (multi) focally by adherent mucoid and semi-solid or dry material that may be crumbling or sloughing, and/or demarcated mucosal ulcers or depressions. Distinct intestinal dilation may be present. Changes found in intestines with score 1 and 2 may also be present (Fig. 1d-h).

Figure 1 illustrates examples on how intestines were scored in accordance with this system. Other examples are provided in Additional file 1: [22].

### Effect of study variables on frequency of severe intestinal lesions and oocyst excretion levels

Challenged and unchallenged turkeys originating from nine experiments were examined for intestinal lesions at three or five weeks of age (Table 1 and Additional file 2: ‘Data lesions all turkeys’ [22]). Neither severe lesions (score3) nor oocysts in intestinal contents were detected among unchallenged 3-week old turkeys or among 3-week-old *Eimeria meleagrimitis*- and *Clostridium perfringens*-inoculated turkeys examined 97–99 h after *Eimeria meleagrimitis* inoculation ([22], Table 1 and data from experiments 3 and 4 included in Table 2). Due to the influencing effects of experiment, turkey age, hours examined after *Eimeria meleagrimitis*-inoculation and *Eimeria meleagrimitis* dose (see below) on prevalence of turkeys with severe intestinal lesions, further analyses were restricted to data sets with comparable values of such influencing variables.

**Table 1:** Design of the nine experiments in the study

Exp	Birds^1^	Age^2^	EM	*CP*		Hours	No.	No.	
			dose^3^	strain	Days^4^	after EM^5^	NE^6^	score3^6^	Mean score^7^
1	3	17	–	–	–	127/145/169	0/0/0	0/0/0	0.0/0.0/1.0
	3	17	5	–	–		0/0/0	0/0/0	0.0/1.0/1.0
	3	17	20	–	–		1/0/0	1/0/0	3.0/1.0/1.0
	15	17	5	04	3,4,5		1/0/0	2/0/0	1.8/1.0/1.0
	15	17	20	04	3,4,5		3/0/0	3/0/0	2.2/1.0/1.4
	15	17	5	13	3,4,5		1/0/0	1/0/0	1.4/1.0/1.0
	15	17	20	13	3,4,5		2/0/1	2/0/1	1.8/1.0/1.4
2	18	17	–	–	–	99/123/145	0/0/0	0/0/0	0.2/0.2/0.3
	18	17	0.25	–	–		0/0/0	0/0/0	0.0/0.8/1.3
	18	17	0.25	04	3,4		0/0/0	0/0/0	0.0/1.3/1.0
	36	17	0.25	04	3,4,5		0/0/0	0/0/0	0.0/1.2/1.3
	18	17	0.25	36	3,4,5		0/0/0	0/0/0	0.2/1.2/1.5
3	22	17	–	–	–	97/121/145/168	0/0/0/0	0/0/0/0	0.2/0.3/0.7/0.5
	18	17	1.60	–	–		0/0/0/−	0/0/0/−	0.3/0.8/0.5/−
	24	17	1.60	04	3,4,5		0/0/1/4	0/0/1/4	0.3/0.6/1.0/3.0
	23	17	1.60	13	3,4,5		0/0/0/0	0/0/0/0	0.2/0.8/1.1/2.0
	24	17	1.60	36	3,4,5		0/0/0/2	0/0/0/2	0.3/0.4/1.2/2.5
4	8	17	–	–	–	99/123/147/171	0/0/0/0	0/0/0/0	0.5/0.5/1.0/0.5
	21	17	5	–	–		0/0/0/0	0/1/0/0	0.5/2.0/1.0/1.0
	20	17	15	–	–		0/2/0/0	0/3/0/0	1.0/2.8/1.0/1.2
	22	17	5	04	3,4,5		0/6/0/1	0/6/0/1	0.8/3.0/1.2/1.5
	23	17	15	04	3,4,5		0/6/0/0	0/6/0/0	1.5/3.0/1.2/1.3
	24	17	5	13	3,4,5		0/6/0/0	0/6/0/0	1.5/3.0/1.2/1.3
	23	17	15	13	3,4,5		0/7/0/0	0/7/0/0	1.0/3.0/1.3/1.0
5	13	17	–	–	–	119/141	0/0	0/0	0.0/0.0
	14	17	5	–	–		0/0	0/0	0.9/1.2
	14	17	5	04	4		0/0	0/0	1.1/1.0
	14	17	5	04	3,4		0/0	0/0	1.0/1.0
	13	17	5	04	3,4,5		0/0	0/0	1.0/0.7
6	18	17	10	04	3,4,5	119/142/168	0/0/0	0/0/0	1.1/1.1/0.0
	17	17	20	04	3,4,5		0/0/0	0/0/0	1.1/1.3/1.3
7	19	15	15	–	–	121/144/168	0/0/0	0/0/0	1.0/1.0/0.5
	21	15	15	04	5,6		1/0/0	1/0/0	1.3/1.1/0.9
	21	15	15	04	4,5,6		0/0/0	0/0/0	1.0/1.1/0.6
8	29	16	10	01	3,4,5,6	124/132/142/149	0/0/0/0	0/0/0/0	0.8/1.0/1.0/0.4
	31	16	10	04	3,4,5,6		0/0/0/0	0/0/0/0	0.8/1.0/1.0/1.0
	31	16	10	13	3,4,5,6		0/0/0/0	0/0/0/0	0.9/1.0/1.0/0.9
	30	16	10	58,702	3,4,5,6		0/0/0/0	0/0/0/0	0.9/0.8/1.0/0.8
9	12	17	10	–	–	125/145	0/0	0/0	1.3/1.0
	12	17	20	–	–		0/0	0/1	1.5/1.6
	12	17	10	04 + 13	3,4,5		0/0	0/0	1.0/1.5
	11	17	20	04 + 13	3,4,5		0/0	1/1	2.3/1.7
	11	30	10	–	–		0/2	2/3	1.5/2.1
	12	30	20	–	–		0/2	2/3	2.0/2.3
	13	30	10	04 + 13	3,4,5		1/7	3/7	2.3/2.8
	12	30	20	04 + 13	3,4,5		2/4	3/6	2.8/2.6

^1^Total number of turkeys/treatment group (row), ^2^Age in days at *Eimeria meleagrimitis*-inoculation, ^3^ Number (in thousands) of *Eimeria meleagrimitis* oocysts inoculated per bird. Strain pM3 was used in experiments 1–8, strain USSMN08–01-5 was used in experiment 9, ^4^ CP = *Clostridium perfringens*, Days = number of days between *Eimeria meleagrimitis*-inoculation and *Clostridium perfringens*-inoculation, ^5^ Number of hours between *Eimeria meleagrimitis*-inoculation and post-mortem examination, ^6^ Number of birds with the appropriate score (NE = mucosal pseudomembrane, ulcer or depression, score3 = score 3 in the scoring system) on each sampling occasion after *Eimeria meleagrimitis*-inoculation, ^7^ Mean score on each sampling occasion

**Table 2:** Effect of sampling time, challenge and age on frequency of severe intestinal lesions

Study variable	Study variable level	CP^1^	EM dose^2^	Hours after EM^3^	Experiments	No. of birds (gut contents)^4^	% score3	OPG^5^
Hrs post	97–99	1	1.6–15	–	3,4	34 (10)	< 3.0^a^	0^a^
EM^6^	121–149	1	1.6–15	–	3,4	91 (20)	29.7^b^	5.8^b^
(3-wk-old)	168–171	1	1.6–15	–	3,4	38 (9)	18.4^ab^	5.0^b^
	123–129	1	5–20	–	1,4,9	53 (16)	64.2^c^	6.3^e^
	145–147	1	5–20	–	1,4,9^7^	59 (10)	1.7^d^	5.4^e^
	125	1	10–20	–	9	8 (2)	12.5^g^	4.6^e^
	145	1	10–20	–	9	15 (2)	6.7^g^	6.2^f^
(5-wk-old)	125	1	10–20	–	9	8 (2)	75.0^c^	4.6^e^
	145	1	10–20	–	9	17 (2)	76.5^c^	6.1^f^
Challenge	None	0	0	119–171	1,2,3,4,5	50 (12)	< 2.1^a^	0^a^
(3-wk-old)	EM^8^	0	0.25–20	119–171	1,2,3,4,5	77 (16)	6.5^a^	5.5^b^
	EM + CP^9^	1	0.25–20	119–171	1,2,3,4,5	278 (43)	15.5^b^	5.6^b^
Turkey age	3-wk-old	1	10–20	125–145	9	23 (8)	8.7^c^	5.8^e^
	5-wk-old	1	10–20	125–145	9	25 (8)	76.0^d^	5.6^e^

^1^*Clostridium perfringens* inoculation (0 = no, 1 = yes) status of lesion scored birds, ^2^ Number (in thousands) of inoculated *Eimeria meleagrimitis* oocysts per bird, ^3^ number of hours elapsed between *Eimeria meleagrimitis* inoculation and examination of turkeys, ^4^ Number of bird scored for intestinal lesions (number of pooled samples of intestinal contents examined for OPG), ^5^ Median log_10_ of oocysts per gram intestinal contents, based on samples of contents from posterior small intestine (from Meckel’s diverticulum to caeco-intestinal junction), caeca and colorectum from *Eimeria meleagrimitis*-inoculated turkeys, ^6^ Hours elapses between *Eimeria meleagrimitis* inoculation and examination for intestinal lesions, ^7^ OPG data from experiments 4 and 9, ^8^ Inoculated with *Eimeria meleagrimitis*, ^9^ Inoculated with *Eimeria meleagrimitis* and *Clostridium perfringens*

^a-b^Dunn’s test with Bonferroni adjustment of *p*-values for multiple comparisons, ^c-d^Pearson chi-square test, ^e-f^Kruskal-Wallis rank test, ^g^Fisher’s exact test. Different letters indicate significant (*p* < 0.05) difference

Three combinations of *Eimeria meleagrimitis* and *Clostridium perfringens* inoculation were included in experiments 1–5 (Tables 1 and 2). Data from these five experiments indicate that a combined *Eimeria meleagrimitis-* and *Clostridium perfringens-*inoculation (EM + CP) of 3-week-old turkeys induced significantly higher prevalence of birds with severe lesions (15.5%) than inoculation with *Eimeria meleagrimitis* alone (EM, 6.5%). The effects on score3 lesions of *Clostridium perfringens* inoculation in *Eimeria meleagrimitis*-inoculated turkeys at three and five weeks of age are displayed in Table 3, experiments 1, 4, 7 and 9. The data indicate a significant increase in percentage of severe lesions from 41.7 to 76.0% in 5-week-old turkeys, corresponding to almost a doubling of prevalence associated with *Clostridium perfringens* inoculation. The percentage increase associated with *Clostridium perfringens* inoculation in 3-week-old turkeys was of similar order, but statistically non-significant. *Clostridium perfringens* inoculation had no significant association with OPG counts (Tables 2 and 3 and Additional file 3: ‘Data OPG.xlsx’ [22]).

**Table 3:** Effect of *Clostridium perfringens* and *Eimeria meleagrimitis* on frequency of severe intestinal lesions

Study variable	Study variable level	EM dose^1^	Hours after EM^2^	Experiments	No. of birds (gut contents)	% score3	OPG^3^
CP^4^	No	10–20	125–145	1,4,7,9^5^	33 (6)	6.1^c^	5.8^e^
3-wk-old	Yes	10–20	125–145	1,4,7,9^5^	57 (8)	12.3^c^	6.0^e^
	No	10–20	125–145	9	24 (4)	4.2^c^	5.8^e^
	Yes	10–20	125–145	9	23 (4)	8.7^c^	5.5^e^
5-wk-old	No	10–20	125–145	9	24 (4)	41.7^c^	5.3^e^
	Yes	10–20	125–145	9	25 (4)	76.0^d^	5.7^e^
EM dose^6^	0.25–1.6	–	121–168	2,3	101 (21)	6.9^a^	5.6^a^
(thousands)	5	–	123–171	1,4	68 (15)	25.0^b^	5.4^a^
	15–20	–	123–171	1,4	68 (15)	27.9^b^	5.5^a^
Low dose	0.25–1.6	–	121–123	2,3	44 (9)	< 2.3^a^	4.5^a^
dynamics		–	145	2,3	45 (9)	2.3^a^	6.1^b^
		–	168	3	12 (3)	46.2^b^	6.2^b^
High dose	5–20	–	123–129	1,4	45 (12)	73.3^a^	6.5^a^
dynamics		–	145–147	1,4	44 (6)^7^	< 2.3^b^	5.2^b^
		–	169–171	1,4	47 (12)	6.4^b^	4.9^b^

^1^Numbers (in thousands) of inoculated *Eimeria meleagrimitis* oocysts per bird, ^2^ Number of hours elapsed between *Eimeria meleagrimitis* inoculation and examination of turkeys, ^3^Median log_10_ of oocysts per gram intestinal contents, based on samples of contents from posterior small intestine (from Meckel’s diverticulum to caeco-intestinal junction), caeca and colorectum from *Eimeria meleagrimitis*-inoculated turkeys ^4^*Clostridium perfringens* inoculation, ^5^ OPG counts based on data from experiments 1, 7 and 9, ^6^ 3-week-old *Eimeria meleagrimitis*-inoculated and *Clostridium perfringens*-inoculated turkeys, ^7^ OPG data only from experiment 4

^a-b^Dunn’s test with Bonferroni adjustment of *p*-values for multiple comparisons. Different letters indicate significant (*p* < 0.05) difference, ^c-d^Pearson chi-square test. Different letters indicate significant (*p* < 0.05) difference, ^e^Kruskal-Wallis rank test. Different letters indicate significant (*p* < 0.05) difference

*Eimeria meleagrimitis* and *Clostridium perfringens* challenged turkeys were examined for frequency of severe intestinal lesions at different time points after *Eimeria meleagrimitis* inoculation (Table 2). Severe intestinal lesions as well as excreted oocysts were detected in *Eimeria meleagrimitis*- and *Clostridium perfringens*-inoculated turkeys examined 121–171 h after *Eimeria meleagrimitis* inoculation (Tables 2 and 4). In 3-week-old turkeys inoculated with 1.6 to 15 thousand oocysts the prevalence of birds with severe lesions was similar in birds examined at 121–149 h and 168–171 h (Table 2, data from experiments 3 and 4). Among 3-week-old turkeys inoculated with 5–20 thousand oocysts, severe lesions were clearly more common at 123–129 h than at 145–147 h after *Eimeria meleagrimitis* inoculation (Table 2, data from experiments 1, 4 and 9). In 5-week-old turkeys inoculated with 10–20 thousand oocysts, the frequencies of birds with severe lesions were high and similar at about 125 and 145 h after *Eimeria meleagrimitis* (Table 2, data from experiment 9).

**Table 4:** Frequency of turkeys with severe intestinal lesions and OPG counts

Experiment (turkey age)	EM dose^1^	Hours after EM^2^	No. of scored birds (samples of intestinal contents)	Percent score3^3^	Median log_10_ OPG^4^
1	5–20	127–129	22 (6)	40.9^c^	6.3^a^
(3-wk-old)		145	22 (−)	< 4.5^d^	–
		169	22 (6)	4.5^d^	5.3^b^
2	0.25	123	24 (5)	< 4.2^a^	5.0^a^
(3-wk-old)		145	24 (5)	< 4.2^a^	5.6^a^
3	1.6	121	20 (4)	< 5.0^c^	0^c^
(3-wk-old)		145	20 (4)	5.0^c^	6.4^d^
		168	13 (3)	46.2^d^	6.2^cd^
4	5–15	123	25 (6)	100.0^c^	6.5^a^
(3-wk-old)		147	24 (6)	< 4.2^d^	5.2^b^
		171	27 (6)	7.4^d^	4.7^b^
5	5	119	23 (4)	< 4.3^a^	4.8^a^
(3-wk-old)		141	18 (4)	< 5.6^a^	6.0^b^
6	10–20	119	17 (2)	< 5.9^c^	3.7^c^
(3-wk-old)		142	13 (2)	< 7.7^c^	6.0^c^
		168	5 (2)	< 20.0^c^	6.0^c^
7	15	121	14 (3)	7.1^c^	6.2^c^
(3-wk-old)		144	14 (3)	< 7.1^c^	5.8^c^
		168	14 (3)	< 7.1^c^	5.9^c^
8	10	124–132	64 (16)	< 1.6^a^	5.8^a^
(3-wk-old)		142–149	57 (16)	< 1.8^a^	5.9^a^
9	10–20	125	8 (4)	12.5^a^	4.6^a^
(3-wk-old)		145	15 (4)	6.7^a^	6.2^b^
(5-wk-old)		125	8 (4)	75.0^a^	4.6^a^
		145	17 (4)	76.0^a^	6.1^b^

^1^Numbers of *Eimeria meleagrimitis* oocysts (in thousands) inoculated per bird, ^2^ Number of hours elapsed between *Eimeria meleagrimitis* inoculation and examination of turkeys, ^3^*Eimeria meleagrimitis*- and *Clostridium perfringens*-inoculated birds, ^4^*Eimeria meleagrimitis*-inoculated birds

^a-b^Kruskal-Wallis rank test. Different letters indicate significant (p < 0.05) difference, ^c-d^ Dunn’s test. Different letters indicate significant (p < 0.05) difference

The peak frequency of severe lesions in experiments based on 3-week-old turkeys varied widely (Table 4). A peak prevalence of at least 40% among 3-week-old turkeys was achieved in experiments 1 and 4, in birds inoculated with 5–20 thousand oocysts and examined 123–129 h after *Eimeria meleagrimitis* inoculation. A high (46.2%) prevalence of severe lesions was also found in 3-week-old turkeys challenged with 1.6 thousand oocysts, in this case at about 168 h after *Eimeria meleagrimitis* inoculation (Table 4, experiment 3).

Experiment 9 was devised to examine the effect of challenge at two different ages of the turkeys (Tables 2 and 4). The impact of *Eimeria meleagrimitis* dose and hours after *Eimeria meleagrimitis* inoculation was controlled for through design. The results indicate a significant and strong (roughly eight to nine times increased frequency at five weeks of age) impact of turkey age on percentage of severe lesions (Table 2). There was no clear difference in prevalence of birds with severe lesions between turkeys examined 125 and 145 h after *Eimeria meleagrimitis* inoculation, although OPG counts differed significantly with sampling time (Table 4). OPG counts did not differ between three and five week old birds.

The effect of *Eimeria meleagrimitis* dose on percentage of severe intestinal lesions was examined in two ways; (a) overall effect of three *Eimeria meleagrimitis* dose levels (sampling 121–171 h after Eimeria meleagrimitis) and (b) the interplay between *Eimeria meleagrimitis* dose level and number of hours after *Eimeria meleagrimitis* inoculation (Table 3). The potential impact of experiment could not be controlled for, because the lowest levels of *Eimeria meleagrimitis* dose (250 and 1600 oocysts per bird) were employed only in experiments 2 and 3 and the highest levels (5000 to 20,000 oocysts) were not tested in these two experiments. Data on overall impact of *Eimeria meleagrimitis* dose indicated similar levels of severe lesions in 3-week-old turkeys inoculated with 5000 and 15–20,000 oocyst, and significantly higher frequencies of severe lesions in such turkeys as compared to turkeys inoculated with 250 to 1600 oocysts. Overall OPG counts were not significantly influenced by *Eimeria meleagrimitis* dose (Table 3). Whereas high (5–20 thousand oocysts) *Eimeria meleagrimitis* doses were associated with early (123–129 h) peaks in OPG counts and severe lesions (Tables 3 and 4, experiments 1 and 4), a low *Eimeria meleagrimitis* dose level (1600 oocysts) was associated with a late (168 h) peak in prevalence of birds with severe lesions (Tables 3 and 4, experiment 3).

A total of 18 (56%) of the 32 turkeys recorded with severe (score 3) lesions in experiment 9 had a macroscopic appearance suggestive of pseudomembranous material adherent to the intestinal mucosa or mucosal ulcers similar to those characteristic of necrotic enteritis lesions in chickens. All 18 cases were detected among 5-week-old *Eimeria meleagrimitis*- inoculated turkeys. The highest prevalence of severe lesions (76%) and classic NE lesions (pseudomembrane/ulcer/depression, 56%) was found in 5-week-old turkeys that were inoculated with *Clostridium perfringens*. No classic NE lesions were present among 3-week-old turkeys from experiment 9 (Table 5). However, such lesions were detected in 9.2% of 3-week-old turkeys that had been inoculated with *Eimeria meleagrimitis* and *Clostridium perfringens* and were examined 123–171 h after *Eimeria meleagrimitis* inoculation ([22], see e.g. Fig. 1F and H).

**Table 5:** Comparison of statistical power of three different macroscopic outcome variables^1^

Group	Turkey age	*Clostridium perfringens* inoculation	No. of turkeys	Median (mean) lesion score	% severe lesions	% pseudomembrane or ulcer/depression
0	3 weeks	No	24	1.5^a^ (1.33)	4.2^a^	< 4.2^a^
1		Yes	23	2.0^ab^ (1.61)	8.7^ab^	< 4.4^a^
2	5 weeks	No	23/24^2^	2.0^bc^ (2.04)	41.7^b^	16.7^a^
3		Yes	25	3.0^c^ (2.64)	76.0^c^	56.0^b^

^1^Median score, percentage of birds with severe lesions and percentage of birds with pseudomembrane/ulcer/mucosal depression as outcome variables. Four combinations of turkey age (3-week-old or 5-week-old) and *Clostridium perfringens* inoculation (with or without *Clostridium perfringens* inoculation) as study variable. Data from experiment 9. All birds were *Eimeria meleagrimitis*-inoculated (10 or 20 thousand oocysts per turkey), ^2^ Data from 23 turkeys for ‘score’ variable (missing value for one turkey), and from 24 turkeys for ‘% severe lesions’ and ‘% pseudomembrane or ulcer/depression’

^a-c^Dunn’s test for multiple comparisons. Different letters indicate significant (p < 0.05) difference

### Comparison of statistical power of outcome variables

Median lesion score, percent severe lesions and percent mucosal pseudomembranes/ulcers/depressions were compared as outcome variables, based on data from experiment 9 (Table 5 and Additional file 4: ‘Data experiment 9’ [22]). All three outcome variables indicated that 5-week-old turkeys inoculated with *Clostridium perfringens* had more pronounced intestinal lesions than the other three groups, although this difference was significant only for percent severe lesions and percent pseudomembranes/ulcers/depressions.

Median lesion scores showed a numeric increase from group 0 to 3. The numeric differences between turkeys with and without *Clostridium perfringens*-inoculation (within age group) were not statistically significant, but the impact of age was significant in turkeys with and without *Clostridium perfringens*-inoculation.

The percentage of severe lesions showed a more marked increase than median lesion score from group 0 to 3, and in this case *Clostridium perfringens* inoculation had a significant impact in 5-week-old turkeys. This lesion type also distinguished between 5-week-old and 3-week-old turkeys, whether they were inoculated with *Clostridium perfringens* or not.

Pseudomembranes/ulcers/depressions distinguished between 5-week-old turkeys that were inoculated with *Clostridium perfringens* or inoculated with *Eimeria meleagrimitis* alone, but did not differentiate between the three other combinations of turkey age and *Clostridium perfringens* inoculation.

### Adverse events and baseline data prior to treatment and testing

The turkeys were clinically healthy before challenge in all but one experiment. In experiment 8 there was increased mortality during the first half of week 2 with a peak on day 9. The cause was possibly a combination of two factors; (a) a change from bell drinkers to nipple drinkers on day 6 may have reduced water uptake temporarily and (b) the sodium contents of the feed was higher than recommended by the breeder company (0.47 vs 0.17%). A new batch of feed was offered as from day 9.

OPG of freshly voided faeces was measured on the day before *Eimeria meleagrimitis* challenge in experiments 1, 4, 6, 7, 8 and 9, with negative results.

Clinical findings following challenge was recorded in experiments 1 and 4; a few challenged birds vomited in association with handling during second or (most often) third *Clostridium perfringens* inoculation.

## Discussion

The ideal outcome variable in an in vivo model of turkey necrotic enteritis would be 100% specific for necrotic enteritis and possible to record without killing or harming the bird. Such an outcome variable is hardly achievable at present, but macroscopic intestinal lesions specific for necrotic enteritis would be a useful substitute. However, the nature of such lesions in turkeys has not been defined. Macroscopic intestinal ulcers, mucosal depressions and mucosal necrosis/pseudomembranes are considered specific for necrotic enteritis in chickens [17, 20, 21]. In spite of this, many studies of experimental necrotic enteritis in chickens employ less specific lesions as part of their scoring system, often with four to seven degrees of severity [10] and not always with a clear definition of the characteristics of each degree. Although a common standard system of lesion scoring in chickens has been proposed [17] and would be very useful, such a system has not yet been established [10]. A contribution towards a common scoring system would be that each grade in the scoring system is clearly defined with a characteristic property. A major hindrance to creating such definitions is probably the fact that not only the severity but also the extent of lesions often is highly variable. Scoring each bird according to the most severe lesion type but not according to extent of lesions is one way of simplifying this task. Another challenge is the variation in lesion severity between experiments and also between sampling occasions within each experiment. This problem could be substantially reduced if the scoring was based on photographs instead of records made during necropsy. Such a procedure is not without drawbacks (in particular the lack of possibility to manipulate the intestine and view the lesions from different angles at the moment of scoring), but these drawbacks are probably minor as compared to the advantage of being able to compare birds across sampling occasions and experiments as a means of calibration. Furthermore, if consensus-based scoring systems are established for necrotic enteritis (in turkeys as well as in chickens), published photographs based on such systems can be used to calibrate scoring practices of different studies and research groups. In this study we have developed and used a scoring system following the above-mentioned proposed approaches, and we have at the same time taken into account the fact that necrotic enteritis-specific lesions have not yet been defined in turkeys.

Scoring systems should produce data allowing for useful statistical analyses. Two main types of outcome variables have been used to measure impact of study variables on necrotic enteritis in chickens; mean/median lesion score and frequency of specific lesion types (e.g. percentage of individuals with necrotic enteritis-specific lesions). In this study, we have focused on a frequency-based outcome, but we have also included an analysis based on median lesion scores as outcome, for comparison with two frequency-based outcome variables (percentage of score 3 lesions and percentage of pseudomembranes/ulcers/depressions). The scoring system developed in this study includes well-defined degrees based largely on villus morphology, and at least one (score 1) and possibly two (scores 1 and 2) scores likely to be associated with *Eimeria meleagrimitis* but not *Clostridium perfringens*. Percentage of pseudomembranes/ulcers/depressions is an outcome considered most likely to be associated with *Clostridium perfringens* based on published literature on chicken NE, but based on published papers on lesions in turkeys associated with *Eimeria meleagrimitis* [e.g. 18] this agent cannot be excluded as a causative factor of such lesions. Score 3 lesions is the most severe lesion degree of our newly developed scoring system, and includes pseudomembranes/ulcers/depressions as well as other types of lesions. The comparison of these three outcome variables (Table 5) suggested that percentage of score 3 (severe) lesions was the most useful regarding statistical discrimination based on analysed data. This outcome variable gave unique *p*-value based superscripts (^a-c^) to three of four combinations of *Clostridium perfringens* inoculation and age, whereas the two other outcomes gave unique superscripts to two study variable combinations. Frequency of score 3 lesions might therefore be useful in studies on experimental necrotic enteritis in turkeys in general.

A main aim of this study was to evaluate the role of factors that might be important to take into consideration in the design of a challenge model of necrotic enteritis in turkeys. Two types of such factors were evaluated statistically; (a) causative factors or factors with a likely influencing effect on necrotic enteritis and (b) a factor that was likely to be important with regard to detection of necrotic enteritis occurrence in the birds. Potentially causative or influencing factors that were evaluated included *Clostridium perfringens* inoculation (yes/no), *Eimeria meleagrimitis* inoculation (yes/no), *Eimeria meleagrimitis* dose (ranging from 0.25 to 20 thousand oocysts) and turkey age (3 or 5 weeks). A factor affecting the likelihood of detecting necrotic enteritis occurrence was sampling time after *Eimeria meleagrimitis* inoculation (ranging from 97 to 171 h).

Our data indicate the importance of combining *Eimeria meleagrimitis* and *Clostridium perfringens* (3–5 days after *Eimeria meleagrimitis*) inoculation as a means of increasing the frequency of turkeys with severe intestinal lesions (score 3). *Clostridium perfringens* inoculation had no statistically significant impact on OPG counts suggesting that *Clostridium perfringens* did not affect parasite replication; this observation supports the assumption that severe lesions were caused principally by *Clostridium perfringens*.

Sampling time after *Eimeria meleagrimitis* and *Clostridium perfringens* inoculation was an important predictor of frequency of turkeys with severe lesions. Sampling four days (97–99 h) after *Eimeria meleagrimitis* inoculation was obviously too early. Although severe intestinal lesions were detected as early as 121 h after *Eimeria meleagrimitis* inoculation, our data [22] suggest that sampling earlier than 123 h after *Eimeria meleagrimitis* implies a risk of missing many birds with imminent lesion development. In 3-week-old turkeys inoculated with five to 20 thousand oocysts, the highest prevalence of birds with severe lesions were found 123–129 h after *Eimeria meleagrimitis* inoculation, but in 5-week-old turkeys there was no significant difference in prevalence between birds sampled at 125 and 145 h after *Eimeria meleagrimitis* (Table 2). For practical reasons we did not examine turkeys between 132 and 141 h after *Eimeria meleagrimitis* inoculation, but our data suggest that this time window comprised peak or near-peak frequencies of birds with severe lesions among 5-week-old turkeys inoculated with 10 to 20 thousand oocysts.

The predisposing effect of *Eimeria meleagrimitis* inoculation on frequency of 3-week-old turkeys with severe lesions appeared to depend on *Eimeria meleagrimitis* dose level (Table 3). The prevalence of birds with severe lesions was clearly lower in experiments with low (250 to 1600 oocysts per bird) *Eimeria meleagrimitis* dose levels than in experiments with high (5000 to 20,000 oocysts per bird) dose levels. *Eimeria meleagrimitis* dose also appeared to influence the dynamics of lesion expression and OPG counts in 3-week-old turkeys. High *Eimeria meleagrimitis* dose levels were associated with peak prevalence of birds with severe lesions at 123–129 h while low *Eimeria meleagrimitis* dose levels were associated with peak prevalence at 168 h after *Eimeria meleagrimitis* (Table 3). These data suggest that the timing of peak expression of severe lesions can be manipulated by *Eimeria meleagrimitis* dose, but possibly not without also affecting the frequency of birds with such lesions. The effect of high vs low *Eimeria meleagrimitis* dose level was not examined in 5-week-old turkeys.

Challenge of 3-week-old turkeys was part of the design of all nine experiments. There was variability between experiments in peak prevalence of turkeys with severe intestinal lesions that was not explained by the above mentioned study variables (*Eimeria meleagrimitis* and *Clostridium perfringens* inoculation, *Eimeria meleagrimitis* dose level and Time of sampling after challenge), as can be seen by comparing results from experiments 1, 4, 8 and 9 (Table 4). We suspected that the highly variable prevalence among 3-week-old turkeys could be associated with the age of the birds, e.g. because of variable levels of maternal antibodies at this age. Data from experiment 9 support this assumption, because prevalence of severe lesions was clearly higher among 5-week-old than 3-week-old turkeys in this experiment (Table 2). We considered a potentially confounding role of unexplained variability in intestinal *Eimeria meleagrimitis* proliferation (measured as OPG counts), but the fact that OPG counts of 3- and 5-week old turkeys were similar (Table 2) indicate that this assumption could not be confirmed. In conclusion, our data suggest that *Eimeria meleagrimitis* inoculation at days 15–17 followed by *Clostridium perfringens* inoculation at days 19–22 (i.e. 3-week-old birds) carries with it a substantial risk of low frequencies of severe lesions (Tables 3 and 4) and even lower levels of pseudomembrane/ulcer/depression frequencies. Further work with in vivo models of turkey necrotic enteritis should therefore test the reproducibility of the age effect demonstrated in experiment 9.

Score 3 intestinal lesions were detected in turkeys that were inoculated with *Eimeria meleagrimitis* only, in particular in turkeys examined around five weeks of age (Table 3). A similar impact of poult age on expression of lesions in the upper intestinal tract of *Eimeria meleagrimitis*- but not *Clostridium perfringens*-inoculated turkeys was documented in a recent work performed at the laboratory of one of the co-authors (JRB) [23]. These findings do not preclude the possibility that *Clostridium perfringens* colonization is a precondition for the emergence of all or some subtypes of severe (score 3) lesions. Colonization of the small intestine by environmental *Clostridium perfringens* strains is typical [15] unless the birds are offered sterilized feed and water and kept and managed in an appropriate way in isolators designed for germfree birds. Birds raised on litter as was the case in the present study, are more likely to be colonized than birds reared on a wire floor. As long as our knowledge about the prevalence of virulent environmental turkey *Clostridium perfringens* strains is insufficient [15, 16], a role of environmental *Clostridium perfringens* strains in lesion induction cannot be ruled out.

The aetiology of score 3 lesions is not fully clarified by our data. Data from experiment 9 (Table 5) indicate that the group of turkeys with intestinal pseudomembranes and ulcers also was the group with highest percentage of score 3 lesions, at the same time as groups with lower levels of score 3 lesions had lower prevalence or were devoid of pseudomembranes/ulcers/depressions. These findings support the assumption that not only pseudomembranes/ulcers/depressions, but also other types of score 3 lesions are caused by *Clostridium perfringens* and constitute part of the complete portfolio of intestinal changes found in turkey necrotic enteritis. However, intestinal pseudomembranes in turkeys have been attributed to *Eimeria meleagrimitis* [18], and more work is needed to establish the role of *Clostridium perfringens* in the development of score 3 types of lesions and to clarify features of these severe lesions that are necrotic enteritis-specific.

A scoring system based on clearly defined grades, an optimal time of sampling after challenge, use of photos facilitating calibration of scoring and outcome variables maximising statistical power are all factors contributing to refinement and reduction of the use of turkeys in research on necrotic enteritis. Increased application of at least some of these factors in the further development of necrotic enteritis models in chickens may be useful.

## Conclusions

In this study we have developed a scoring system for use in experimental turkey necrotic enteritis with *Eimeria meleagrimitis* as a predisposing factor. We used this scoring system to demonstrate the impact on macroscopic intestinal lesion induction of *Clostridium perfringens* inoculation, *Eimeria meleagrimitis* inoculation, *Eimeria meleagrimitis* dose level, turkey age and time elapsed between *Eimeria meleagrimitis* challenge and examination of the intestine. This study represents a first and major step forward in the development and use of in vivo experimental models of necrotic enteritis in turkeys, and a basis for acquisition of new knowledge about the pathogenesis, immunity, microbiota and other important aspects of this disease.

## Methods

### Study design

This study can be divided into two phases. The first phase comprised experiments number 1–8 and was based on the examination of 3-week-old turkeys (Table 1). The following study variables were tested: *Eimeria meleagrimitis* inoculation (yes/no), *Eimeria meleagrimitis* dose (250, 1600, 5000, 10,000, 15,000, 20,000 oocysts per turkey), *Clostridium perfringens* inoculation (yes/no) and number of hours elapsed between *Eimeria meleagrimitis* inoculation and examination of the turkeys (varied from 97 to 171 h). Three combinations of *Eimeria meleagrimitis* and *Clostridium perfringens* challenge were tested during phase 1: (a) turkeys with neither *Eimeria meleagrimitis* nor *Clostridium perfringens* challenge, (b) turkeys inoculated with *Eimeria meleagrimitis* alone and (c) turkeys inoculated with *Eimeria meleagrimitis* and subsequently with *Clostridium perfringens*.

The second phase comprised experiment number 9, with testing of the following four binary study variables: *Clostridium perfringens* inoculation (yes/no), *Eimeria meleagrimitis* dose (10,000 or 20,000 oocysts per turkey), turkey age (three or five weeks at necropsy) and number of hours elapsed between *Eimeria meleagrimitis* inoculation and examination (125 or 145 h).

Sample size estimation was based on the assumption that a frequency of challenged individuals would show severe macroscopic intestinal lesions (primary experimental outcome) that were absent in unchallenged individuals. Frequency of lesions was likely to be associated with time of examination after challenge. Because optimal time point for examination was unknown, we examined birds on two to four occasions per experiment. The sampsi procedure of Stata 13 was used to explore sample sizes based on a significance level 0.05 and a power of 0.90. These calculations indicated sample sizes varying from 5 to 47 depending on expected lesion frequency (90–20%) in the positive control group and whether the test was one-sided or two-sided. Although higher statistical power would have been desirable, we decided to design the study with sample sizes per time point and experiment that varied between five and eight in most treatment groups (Table 1), corresponding to 90–75% occurrence of severe intestinal lesions in the positive control group. The statistical power was increased by merging treatments groups within experiments (Tables 4 and 5, and Table 2: Turkey age). Furthermore, because we conducted a total of nine experiments, we could increase sample size by merging observations from identical treatments in different experiments (Tables 2 and 3).

### Animals, housing and feed

Day-old male turkeys (B.U.T. 10 in experiments 1–8, B.U.T. Premium in experiment 9) were supplied by a commercial hatchery (Baastad Kalkun, 1866 Båstad, Norway). The birds arrived in one or two transportation boxes, and were selected at random for allocation to experimental groups. In each experiment, all birds originated from the same parent flock. One day before challenge, bird size of all treatment groups was compared visually, and birds were moved in order to make the average bird size of each treatment group as equal as possible. All turkeys were kept on floor covered by wood shavings (new at the beginning of each experiment) during the entire experimental period, and were housed either in up to 21 cages (0.300 m^2^) or up to 10 pens (1.135 m^2^). Cages were used in experiments 1–4 and pens were used in experiments 5–9. Maximum estimated total live weight per m^2^ during each experiment was 10.0 kg and 10.9 kg for cages and pens, respectively. Total live weight estimates were based on Commercial Performance Objectives of B.U.T. 10 and B.U.T. Premium (http://www.aviagenturkeys.com/en-gb/documents) as well as recordings of actual live weights. The birds had free access to drinking water and feed, continuous light during the first 24 h after housing and an eight-hours darkness/16-h light cycle during the rest of each experiment.

All turkeys in this study were euthanized, either because of disease/disorders, retarded growth or (in most cases) sampling for data collection. Birds were euthanized by blunt force trauma to the head, to render unconsciousness, followed by cervical dislocation.

Room temperature was monitored daily and adjusted with bird age according to guidelines from the hatchery. No anaesthetics or surgical interventions requiring anaesthetics were used in this study. All experiments, including the method of euthanasia, were approved by the Norwegian Food Safety Authority (FOTS applications ID 5373, 5394 and 10,491).

Experimental feeds were used in experiments 1–7. In experiments 1–6 a starter feed was used until day 15–17, followed by a grower feed until the end of the experiment. In experiment 7 the grower feed was used from day 0 throughout the experiment. Major ingredients of the experimental starter feed were maize, dehulled oats, extracted soybean meal and fishmeal. Major ingredients of the experimental grower feed were wheat, barley, fishmeal, extracted soybean meal and animal fats. In experiments 8 and 9 all birds were offered a single type of commercial feed for meat type poultry. Major ingredients were wheat, oats, extracted soybean meal, maize grits, maize gluten, fishmeal, animal fats and vegetable fats. None of the used feeds were supplemented with any antibiotics or anticoccidial compounds.

### Preparation of inoculum and inoculation

Two strains of *Eimeria meleagrimitis* were used. Only one strain was used in each experiment. Strain pM3 was used in experiments 1–8 and strain USMN08–01-Line 5 was used in experiment 9. Strain pM3 was obtained from a turkey farm in Brittany, France and purified from one single oocyst in 1999. The strain has been maintained at the Ploufragan-Plouzané-Niort Laboratory of Anses (French agency for food, environmental and occupational health and safety) by regular passages in turkeys free from coccidia since it was obtained. Strain USMN08–01-5 was obtained from a turkey farm in Minnesota, USA in 2008, isolated and propagated at the University of Arkansas in Fayetteville, USA, and further characterized at the University of Guelph in Ontario, Canada [18]. Oocysts were kept in potassium dichromate suspension at 4^°^C until the inoculum was prepared within 24 h of use. In experiments 1–7 the oocyst suspension was centrifuged at 1300×g for 10 min, the supernatant with potassium dichromate was removed and the oocysts were resuspended in PBS. Oocyst concentration was then estimated based on counts in a Fuchs Rosenthal chamber, and adjusted by adding PBS if necessary. Suspensions were thoroughly homogenized immediately prior to every step of sampling and dilution. Oocysts with morphologic signs suggesting lack of infectivity/viability were not included in the counts. At least six samples from each dilution were counted. Inoculum in experiments 8 and 9 were prepared in the same way as in experiments 1–7 with the following exceptions: the original suspension was not centrifuged and potassium dichromate was not removed.

A total of five *Clostridium perfringens* isolates (001, 004, 013, 036 and 58,702) were used. All of these isolates were recovered from severe intestinal lesions in commercial Norwegian turkeys diagnosed with necrotic enteritis. Isolate 01 was used in one experiment, isolate 04 in all experiments, isolate 13 in five experiments, isolate 36 in two experiments and isolate 58,702 in one experiment. In experiments 1–8 each *Clostridium perfringens* -challenged bird was inoculated with only one *Clostridium perfringens* isolate. In experiment 9 each challenged bird was inoculated with a mix of isolates 04 and 13.

*Clostridium perfringens* was inoculated into 200 ml pre-reduced and pre-warmed Brain Heart Infusion broth (BHI) and incubated anaerobically at 37^o^ C overnight (15 h) when it was diluted 1 in 2 with fresh BHI. This inoculum was introduced to the birds within 2 h of preparation. *Clostridium perfringens* challenge was done on day three to six after *Eimeria meleagrimitis* inoculation, in most cases (see Table 5) on days 3, 4 and 5 after *Eimeria meleagrimitis* inoculation.

*Eimeria meleagrimitis* and *Clostridium perfringens* inoculates were administered into the crop of each bird, using a flexible plastic tube fitted onto a syringe. Low-dose *Eimeria* inoculation was conducted prior to high-dose inoculation. Hygienic measures were taken to avoid cross-contamination between treatment groups. Control birds were left un-inoculated.

### Sampling, data recording and laboratory analyses

A total of 810 turkeys were moved from the experimental facility and euthanized (see above for details), examined and sampled during varying time periods following *Eimeria meleagrimitis* and *Clostridium perfringens* inoculation. The turkeys were put down and examined at 21 to 36 days of age. The small intestine between the gizzard and Meckel’s diverticulum of all birds was opened and inspected closely for mucosal lesions. If severe lesions were detected in the posterior part of the opened intestinal segment, the unopened part of the small intestine was opened and inspected for lesions. Lesions that were considered severe or suggestive of necrotic enteritis were recorded. At least one digital photograph of the opened intestine of each turkey was taken and used for the development of a scoring system and subsequent final scoring of each bird. The turkeys were examined for intestinal lesions on days 4–7 (about 97–171 h) after *Eimeria meleagrimitis* inoculation, or at corresponding age in non-inoculated turkeys. A majority of the turkeys were examined on day 5 or 6 after *Eimeria meleagrimitis* inoculation, in most cases corresponding to the last day of *Clostridium perfringens* inoculation and the day after (Table 1).

Intestinal contents (posterior small intestine from Meckel’s diverticulum to the caeco-intestinal junction, caeca and colorectum) were pooled from birds that were sampled on same occasion and had identical combination of *Eimeria meleagrimitis* and *Clostridium perfringens* challenge (including *Clostridium perfringens* isolate). Collectively these 152 pooled samples originated from 97.2% (787/810) of all turkeys that were examined for intestinal lesions. The mean number of individuals contributing to each pooled sample was 5.2. OPG counts were calculated based on routine laboratory procedures using a modified McMaster flotation technique and Whitlock Universal McMaster counting chambers.

### Scoring system development and scoring

Development of the macroscopic lesion scoring system was based on written records of necropsy findings and inspection of 2312 photographic images of opened intestines from the examined birds. A four tier scoring system was devised, graded by lesion severity. A score 0 was given to turkeys with a small intestine considered macroscopically normal. A score 1 was given to turkeys with the mildest lesions, and a score 3 was given to turkeys with the lesions considered most severe. Important aspects used to form the four lesion categories include characteristics of the intestinal wall, mucosa, mucosal villi, intestinal contents and materials on the mucosal surface. Scores 1 and 3 were defined by one characteristic lesion type that was not present in birds with lower scores (but could be present in birds with higher scores). Scoring of each turkey was conducted after the scoring system had been established.

### Relationships between study variables and frequency of severe intestinal lesions

The experimental unit was individual turkeys. The primary experimental outcome was recorded as presence or absence of score 3 lesions (a binary variable). Outcome estimates were reported as percentage of examined turkeys with lesion score 3.

The role of six independent variables in potentially influencing the primary outcome variable was examined: Challenge (categorical, 3 combinations of *Eimeria meleagrimitis*/*Clostridium perfringens* inoculation), *Eimeria meleagrimitis* dose (categorical, 2 or 3 levels), *Clostridium perfringens* inoculation (binary), Hours elapsed between *Eimeria meleagrimitis* inoculation and examination (also designated ‘Hours after *Eimeria meleagrimitis* inoculation’) (categorical, 2 to 4 levels), turkey Age (binary) and Experiment (categorical).

The effect of each of these study variables was analysed under best possible control for the potentially influencing effect of the other independent variables (control variables) on the frequency of severe lesions. Such control was achieved by use of subsets of available data, where all control variables were kept constant and similar for each level of the study variable. As an example, the potential impact of Challenge was controlled for by including only turkeys from the same subsets of *Eimeria meleagrimitis* dose, Hours after *Eimeria meleagrimitis* and Experiments (Table 2).

Two-sided Fisher’s exact test or Pearson chi-square test (tabulate procedure, Stata/MP 14.2) were used to compare two groups, while Dunn’s test with Bonferroni adjustment (dunntest procedure, Stata/MP 14.2) was used for multiple comparisons. *P*-values below 0.05 were considered statistically significant.

### Relationships between study variables and oocyst excretion

The experimental unit was pooled samples of intestinal contents from turkey groups defined during the analyses of relationships between study variables and severe intestinal lesions (see above, and Tables 2, 3 and 4). Pooled samples were used to analyse the impact of independent variables on OPG counts. OPG was a secondary (continuous) outcome variable. Oocyst counts were log10-transformed. Outcome estimates were reported as median log_10_ OPG counts.

The distribution of OPG counts within each turkey group was in most cases non-normal (Shapiro-Wilk normality test, swilk procedure in Stata 14.2). OPG data were therefore analysed using non-parametric statistical methods. Kruskal-Wallis rank test (kwallis procedure, Stata/MP 14.2) was used to compare two groups, while Dunn’s test with Bonferroni adjustment (dunntest procedure, Stata/MP 14.2) was used for multiple comparisons. P-values below 0.05 were considered statistically significant.

### Comparison of statistical power of outcome variables

Data from experiment 9 were used. The unit of analysis was individual turkeys. The study variable in this comparison was based on four combinations (designated groups in Table 5) of turkey age and *Clostridium perfringens* inoculation (0 = 3-week-old turkeys without *Clostridium perfringens* inoculation, 1 = 3-week-old turkey with *Clostridium perfringens* inoculation, 2 = 5-week-old turkeys without *Clostridium perfringens* inoculation, and 3 = 5-week-old turkeys with *Clostridium perfringens* inoculation). Three types of outcome variable were compared; frequency of birds with score 3 lesions (severe lesions) per turkey group, frequency of birds with intestinal pseudomembranes/ulcers-depressions per turkey group and median intestinal scores (0–3 as defined in the developed scoring system) per turkey group. Intestines were scored with pseudomembranes if apparently adherent mucoid and semisolid or dry material was present (e.g. as in Fig. 1F). Ulcers/depressions were scored if clearly demarcated mucosal depressions with (ulcers) or without (depressions, see Fig. 1H) haemorrhage were found.

Median rather than mean intestinal scores and non-parametric testing was used because intestinal scores were not normally distributed (Shapiro-Wilk normality test, swilk procedure in Stata/MP 14.2). Dunn’s test with Bonferroni adjustment (dunntest procedure, Stata/MP 14.2) for multiple comparisons was used for analysis of impact of the study variable on all three outcome variables. P-values below 0.05 were considered statistically significant.

## Supplementary information

**Additional file 1.** Additional photos of intestinal lesions. Folder with 47 additional photos in JPG format illustrating turkeys with intestinal lesions that were assigned to scores 0, 1, 2 or 3.

**Additional file 2.** Data supporting conclusions regarding impact of study variables on frequency of turkeys with severe intestinal lesions.

**Additional file 3.** Data supporting conclusions regarding impact of study variables on OPG counts.

**Additional file 4.** Data supporting conclusions on the ability to differentiate statistically between levels of study variables when using three different outcome variables.

## Data Availability

Additional files supporting the conclusions of this article, and a folder containing additional photographic images supporting development of a scoring system for macroscopic intestinal lesions in turkeys are available in the Mendeley Data repository at https://data.mendeley.com/datasets/7mbcpywvpr/draft?a=90a6323f-709a-42ff-9346-15b546d93516. The file ‘Data lesions all turkeys.xlsx’ supports conclusions regarding impact of study variables on frequency of turkeys with severe intestinal lesions, ‘Data OPG.xlx’ supports conclusions regarding impact of study variables on OPG counts, and ‘Data experiment 9.xlsx’ supports conclusions on the ability to differentiate statistically between levels of study variables when using frequency of turkeys with severe lesions, frequency of turkeys with classic necrotic enteritis lesions and intestinal lesion score as outcome variables.

## Abbreviation

OPG: number of *Eimeria* oocysts per gram intestinal contents
