# Supplementary figures and images for: Developing an experimental necrotic enteritis model in turkeys - the impact of Clostridium perfringens, Eimeria meleagrimitis and host age on frequency of severe intestinal lesions
Source: BMC Vet Res. 2020 Feb 18;16:63. doi: 10.1186/s12917-020-2270-5 (PMC7029515; doi:10.1186/s12917-020-2270-5)

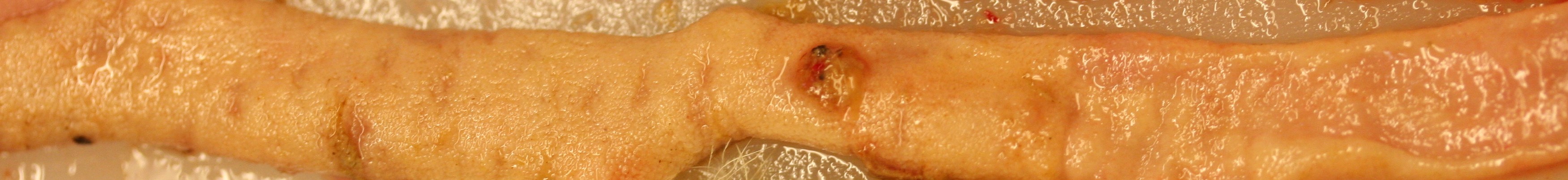

Supplement: Supplementary file 1 — Additional file 1. Additional photos of intestinal lesions. Folder with 47 additional photos in JPG format illustrating turkeys with intestinal lesions that were assigned to scores 0, 1, 2 or 3. [file 12917_2020_2270_MOESM1_ESM.zip › Score 3 Id14 Exp7.jpg]

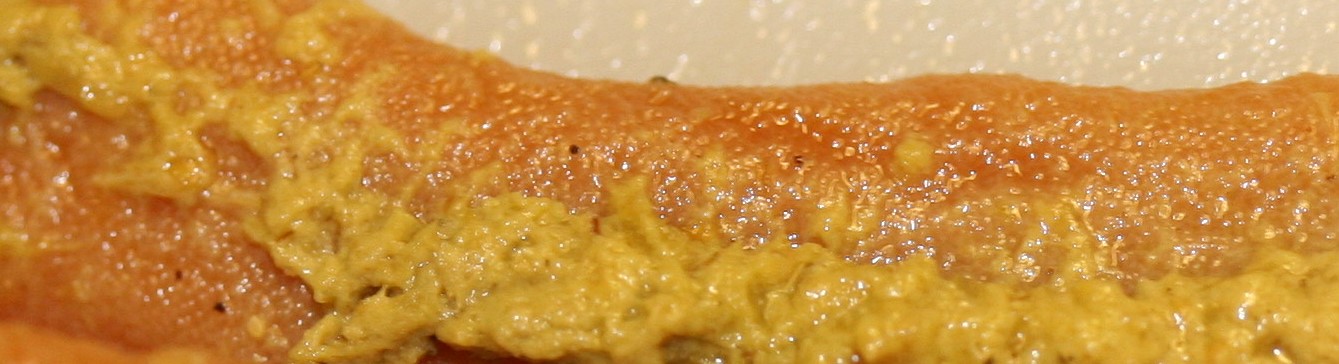

Supplement: Supplementary file 1 — Additional file 1. Additional photos of intestinal lesions. Folder with 47 additional photos in JPG format illustrating turkeys with intestinal lesions that were assigned to scores 0, 1, 2 or 3. [file 12917_2020_2270_MOESM1_ESM.zip › Score0 Id23G Exp1 Duodenum.jpg]

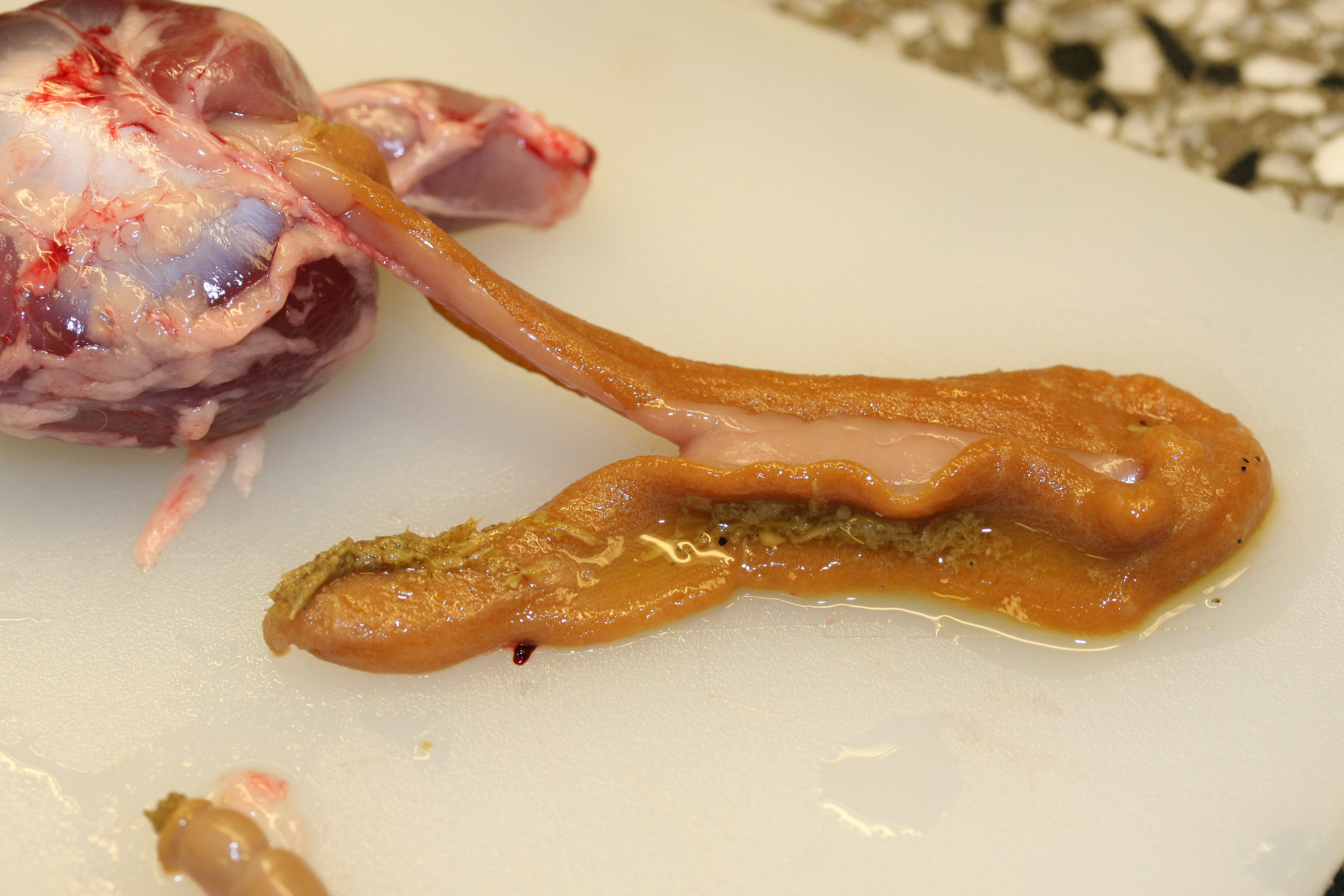

Supplement: Supplementary file 1 — Additional file 1. Additional photos of intestinal lesions. Folder with 47 additional photos in JPG format illustrating turkeys with intestinal lesions that were assigned to scores 0, 1, 2 or 3. [file 12917_2020_2270_MOESM1_ESM.zip › Score0 Id67G Exp1.jpg]

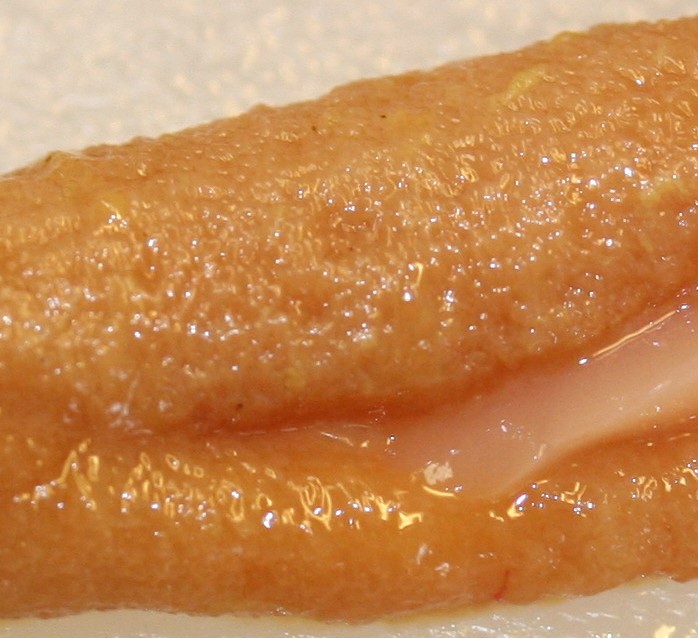

Supplement: Supplementary file 1 — Additional file 1. Additional photos of intestinal lesions. Folder with 47 additional photos in JPG format illustrating turkeys with intestinal lesions that were assigned to scores 0, 1, 2 or 3. [file 12917_2020_2270_MOESM1_ESM.zip › Score0 IdG2 Exp4 Duodenum.jpg]

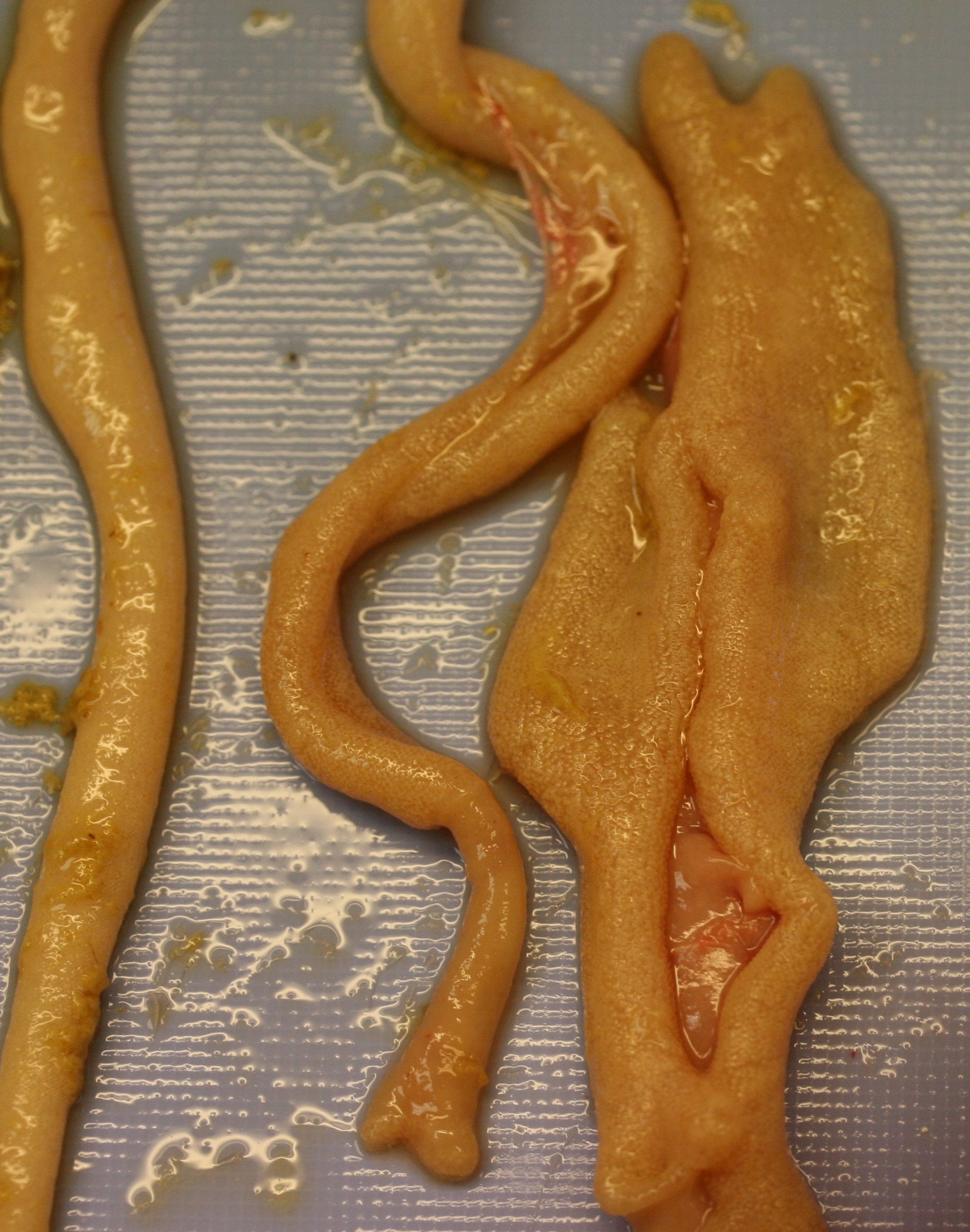

Supplement: Supplementary file 1 — Additional file 1. Additional photos of intestinal lesions. Folder with 47 additional photos in JPG format illustrating turkeys with intestinal lesions that were assigned to scores 0, 1, 2 or 3. [file 12917_2020_2270_MOESM1_ESM.zip › Score1 IdC8 Exp5 .jpg]

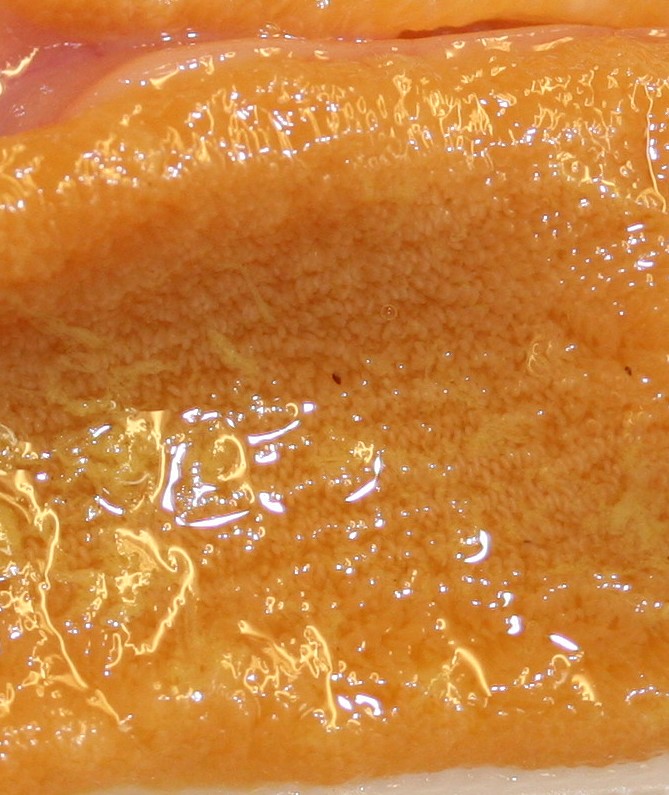

Supplement: Supplementary file 1 — Additional file 1. Additional photos of intestinal lesions. Folder with 47 additional photos in JPG format illustrating turkeys with intestinal lesions that were assigned to scores 0, 1, 2 or 3. [file 12917_2020_2270_MOESM1_ESM.zip › Score1 IdU1 Duodenum.jpg]

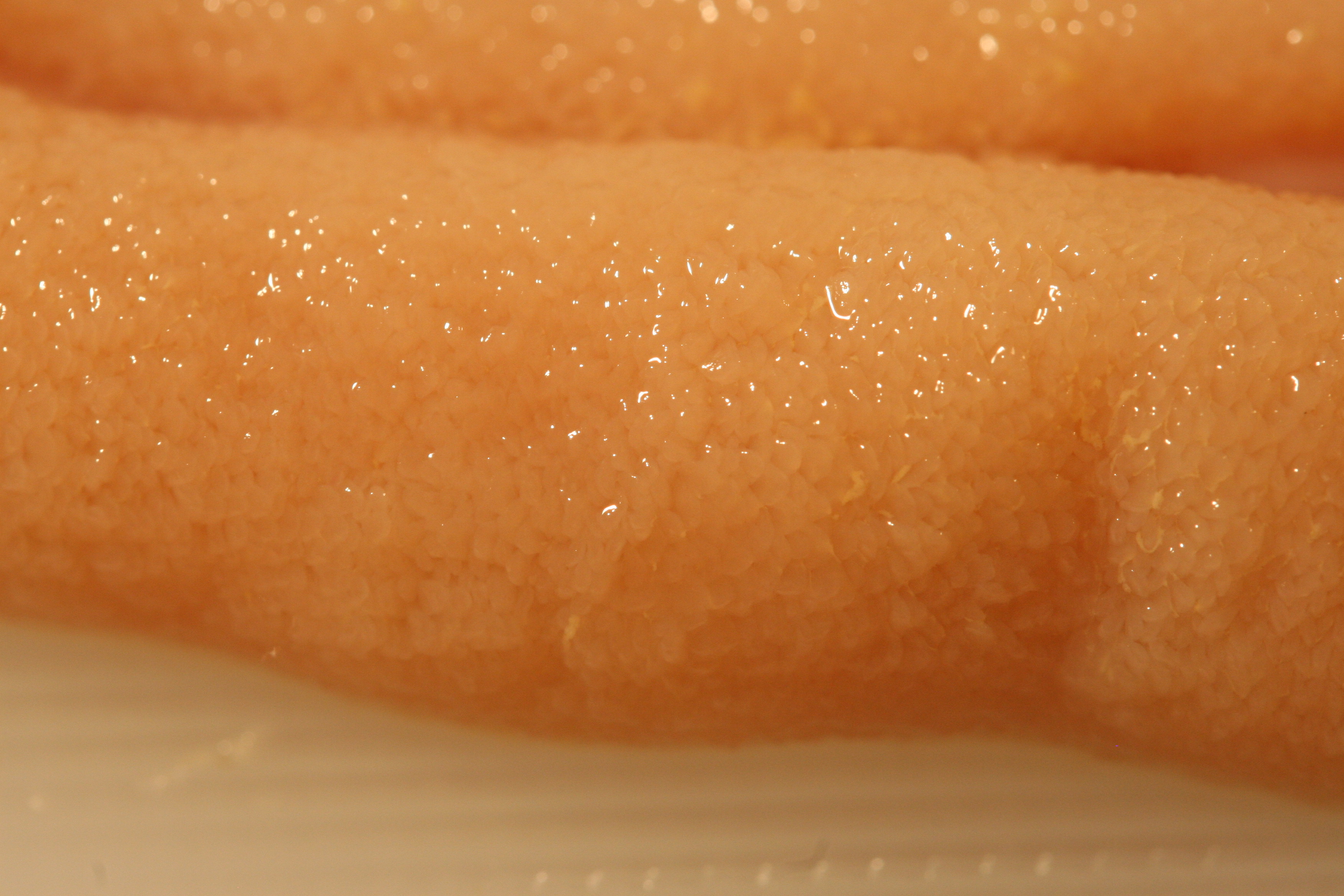

Supplement: Supplementary file 1 — Additional file 1. Additional photos of intestinal lesions. Folder with 47 additional photos in JPG format illustrating turkeys with intestinal lesions that were assigned to scores 0, 1, 2 or 3. [file 12917_2020_2270_MOESM1_ESM.zip › Score1 IMG_3162 P704 49A.jpg]

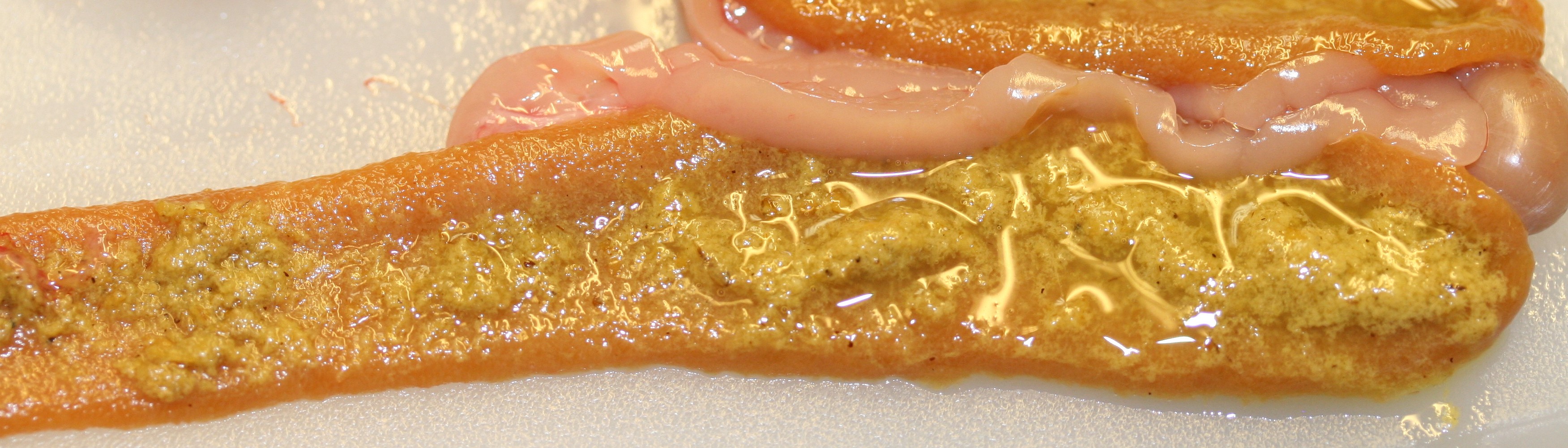

Supplement: Supplementary file 1 — Additional file 1. Additional photos of intestinal lesions. Folder with 47 additional photos in JPG format illustrating turkeys with intestinal lesions that were assigned to scores 0, 1, 2 or 3. [file 12917_2020_2270_MOESM1_ESM.zip › Score1 IMG_4063 Exp3 .jpg]

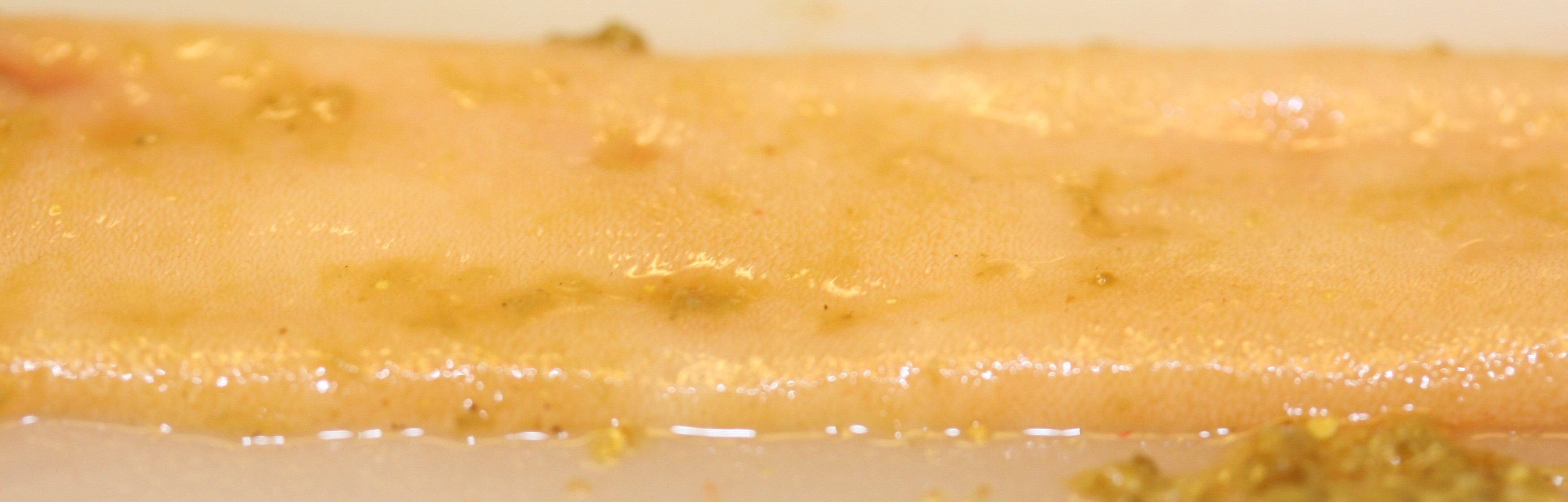

Supplement: Supplementary file 1 — Additional file 1. Additional photos of intestinal lesions. Folder with 47 additional photos in JPG format illustrating turkeys with intestinal lesions that were assigned to scores 0, 1, 2 or 3. [file 12917_2020_2270_MOESM1_ESM.zip › Score1 IMG_4185 Exp3 .jpg]

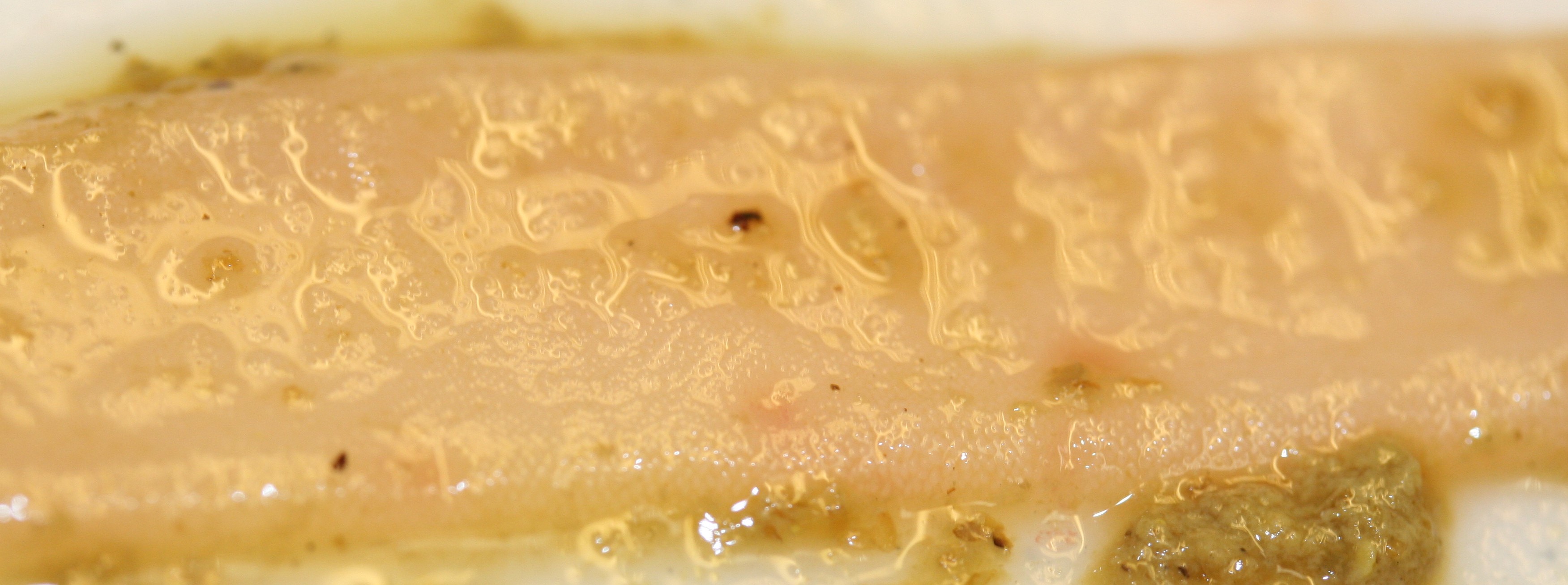

Supplement: Supplementary file 1 — Additional file 1. Additional photos of intestinal lesions. Folder with 47 additional photos in JPG format illustrating turkeys with intestinal lesions that were assigned to scores 0, 1, 2 or 3. [file 12917_2020_2270_MOESM1_ESM.zip › Score1 IMG_4188 Exp3 .jpg]

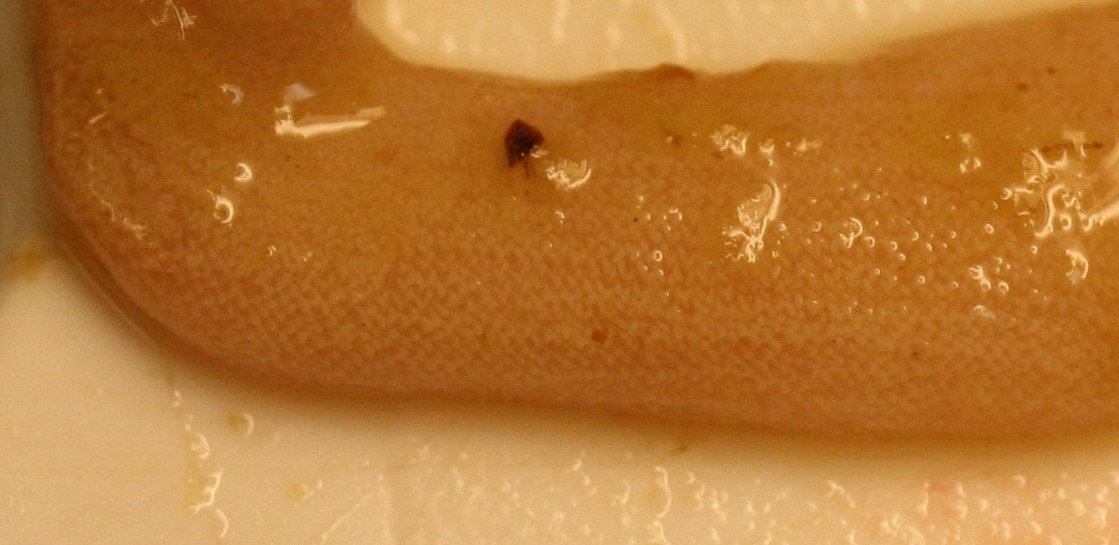

Supplement: Supplementary file 1 — Additional file 1. Additional photos of intestinal lesions. Folder with 47 additional photos in JPG format illustrating turkeys with intestinal lesions that were assigned to scores 0, 1, 2 or 3. [file 12917_2020_2270_MOESM1_ESM.zip › Score1-2 Id14 Exp6.jpg]

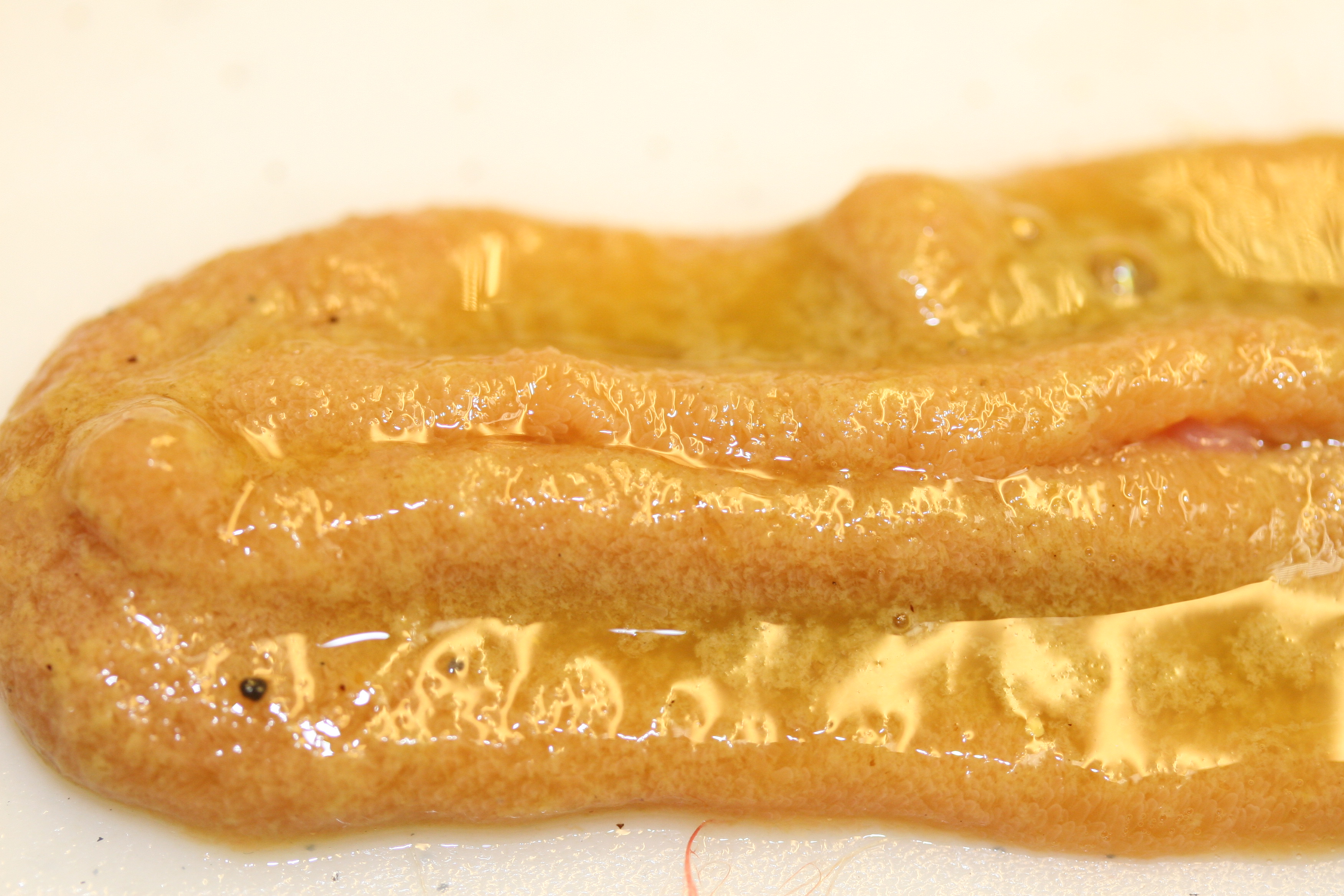

Supplement: Supplementary file 1 — Additional file 1. Additional photos of intestinal lesions. Folder with 47 additional photos in JPG format illustrating turkeys with intestinal lesions that were assigned to scores 0, 1, 2 or 3. [file 12917_2020_2270_MOESM1_ESM.zip › Score1-2 IMG_4200 Exp3.jpg]

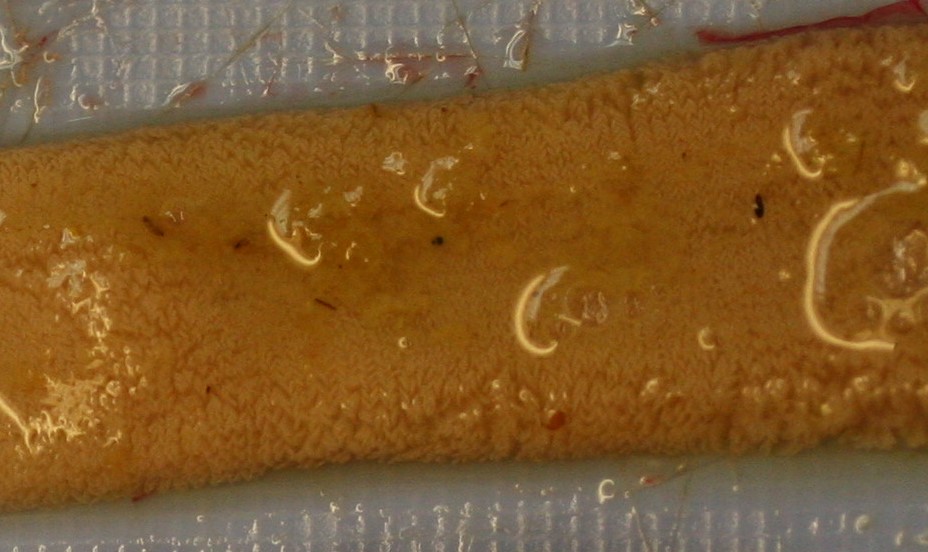

Supplement: Supplementary file 1 — Additional file 1. Additional photos of intestinal lesions. Folder with 47 additional photos in JPG format illustrating turkeys with intestinal lesions that were assigned to scores 0, 1, 2 or 3. [file 12917_2020_2270_MOESM1_ESM.zip › Score2 Id17 Exp7 Jejunum.jpg]

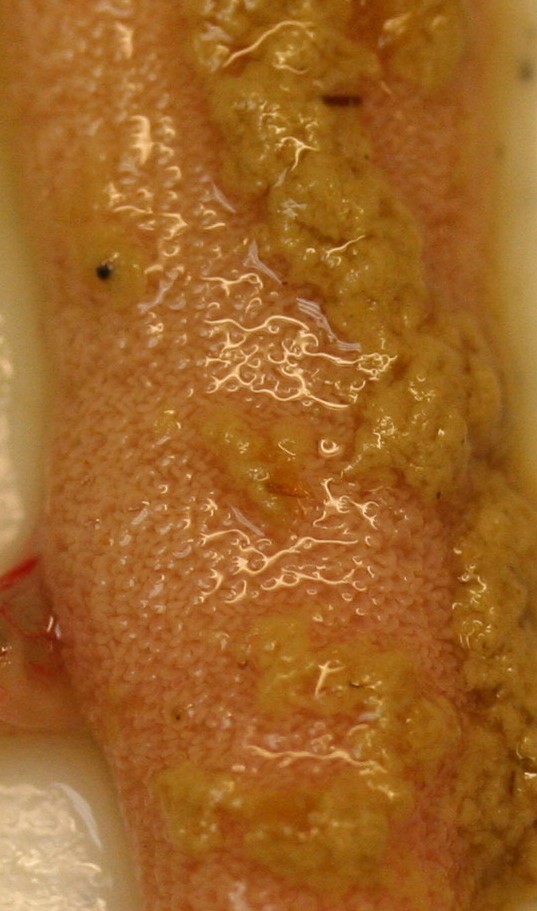

Supplement: Supplementary file 1 — Additional file 1. Additional photos of intestinal lesions. Folder with 47 additional photos in JPG format illustrating turkeys with intestinal lesions that were assigned to scores 0, 1, 2 or 3. [file 12917_2020_2270_MOESM1_ESM.zip › Score2 Id31a Exp6.jpg]

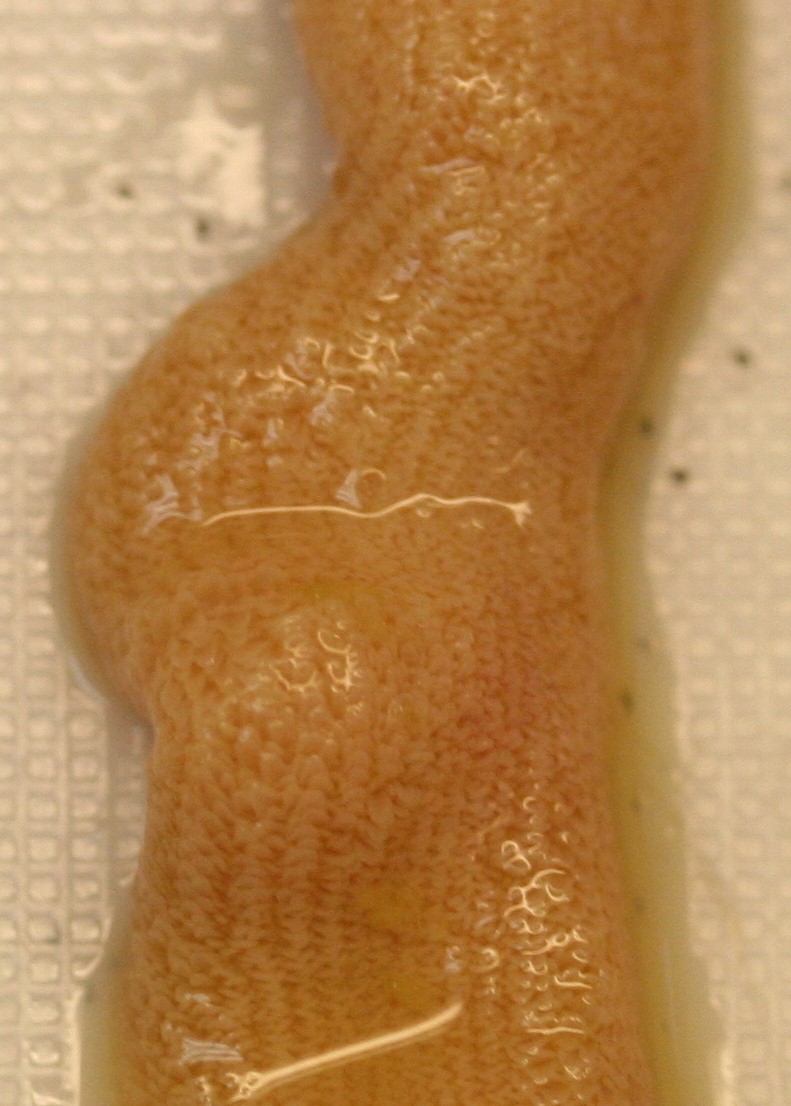

Supplement: Supplementary file 1 — Additional file 1. Additional photos of intestinal lesions. Folder with 47 additional photos in JPG format illustrating turkeys with intestinal lesions that were assigned to scores 0, 1, 2 or 3. [file 12917_2020_2270_MOESM1_ESM.zip › Score2 Id31bExp6.jpg]

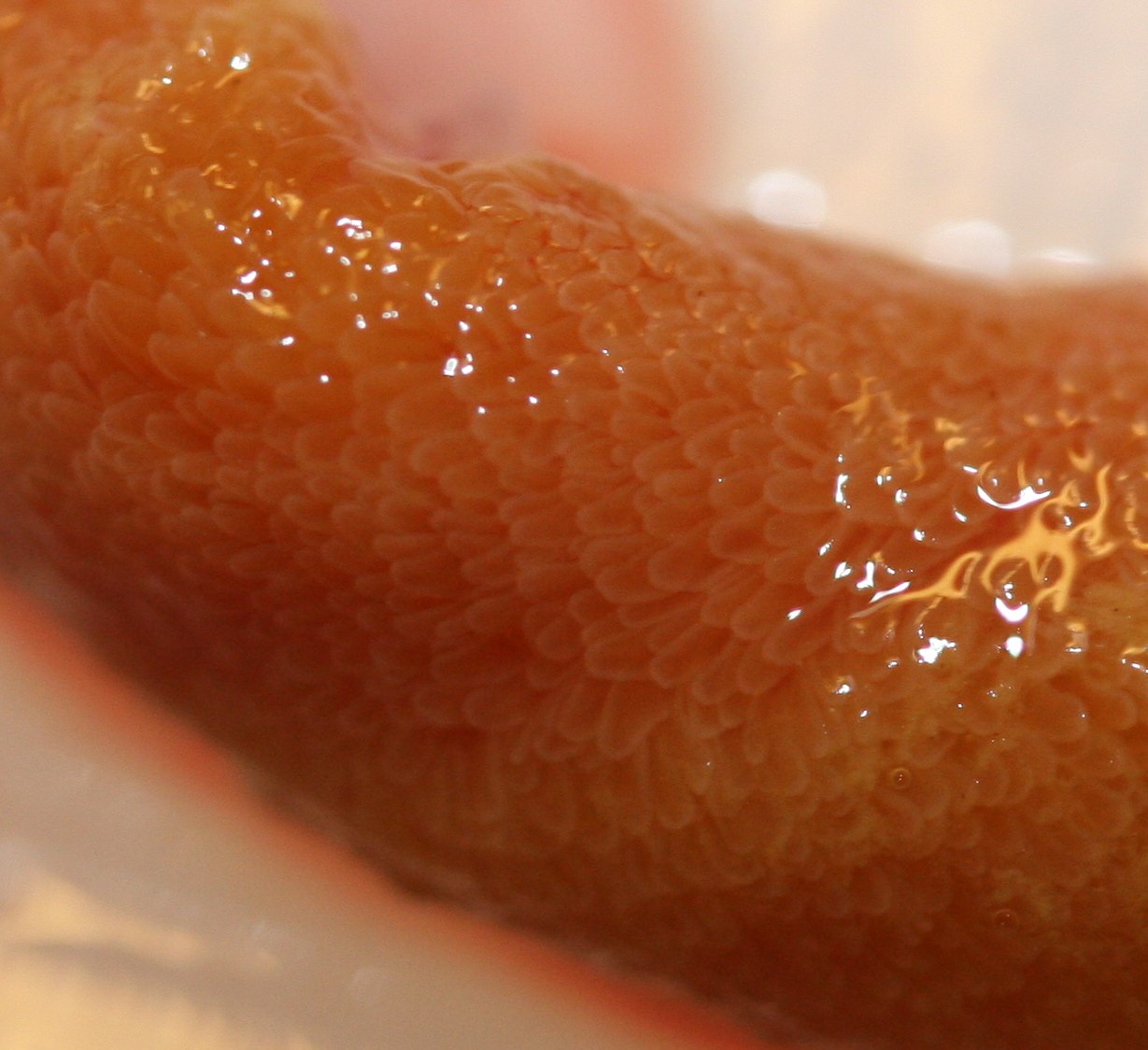

Supplement: Supplementary file 1 — Additional file 1. Additional photos of intestinal lesions. Folder with 47 additional photos in JPG format illustrating turkeys with intestinal lesions that were assigned to scores 0, 1, 2 or 3. [file 12917_2020_2270_MOESM1_ESM.zip › Score2 IdL8 Exp2 .jpg]

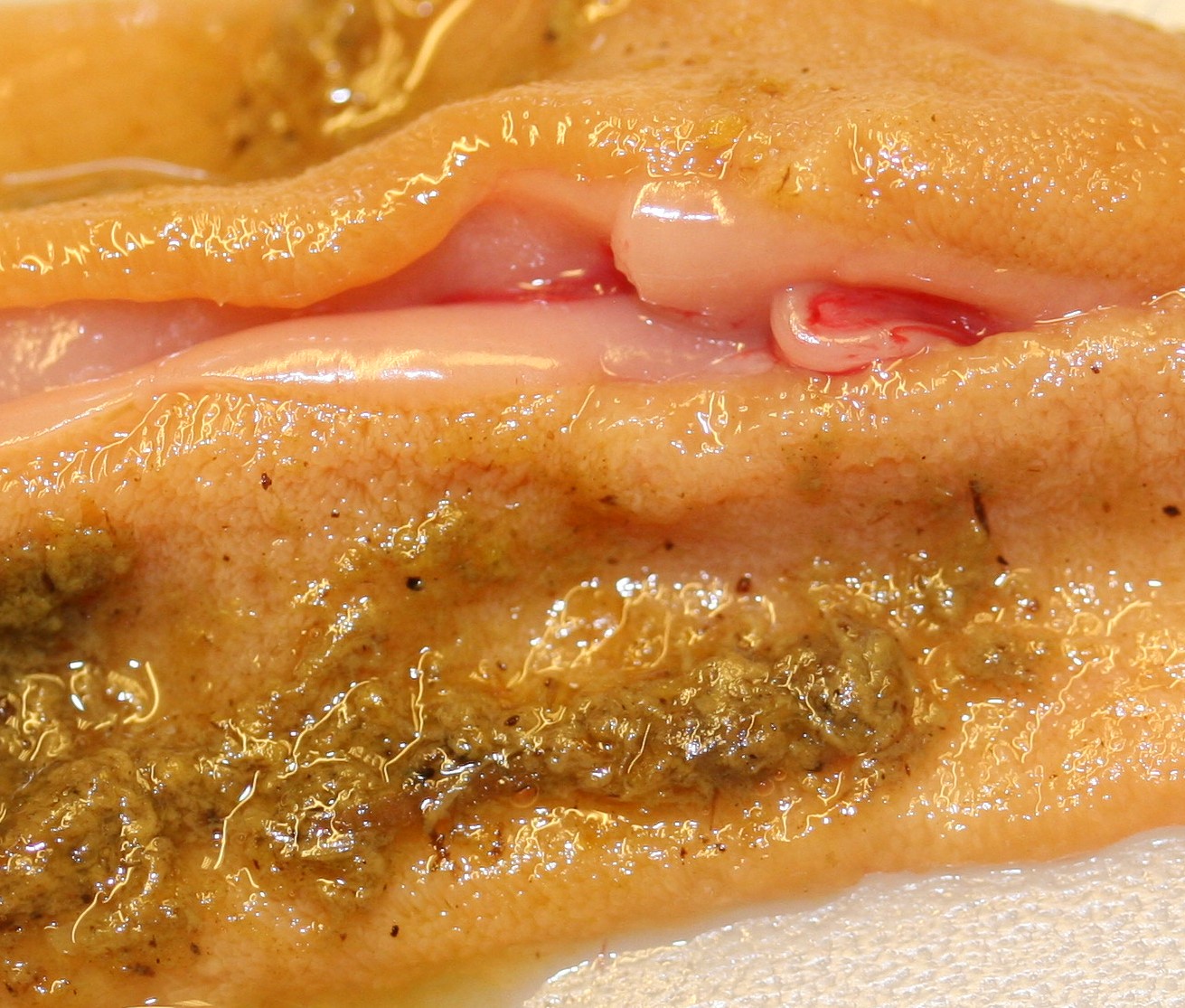

Supplement: Supplementary file 1 — Additional file 1. Additional photos of intestinal lesions. Folder with 47 additional photos in JPG format illustrating turkeys with intestinal lesions that were assigned to scores 0, 1, 2 or 3. [file 12917_2020_2270_MOESM1_ESM.zip › Score2 IdQ3 Duodenum.jpg]

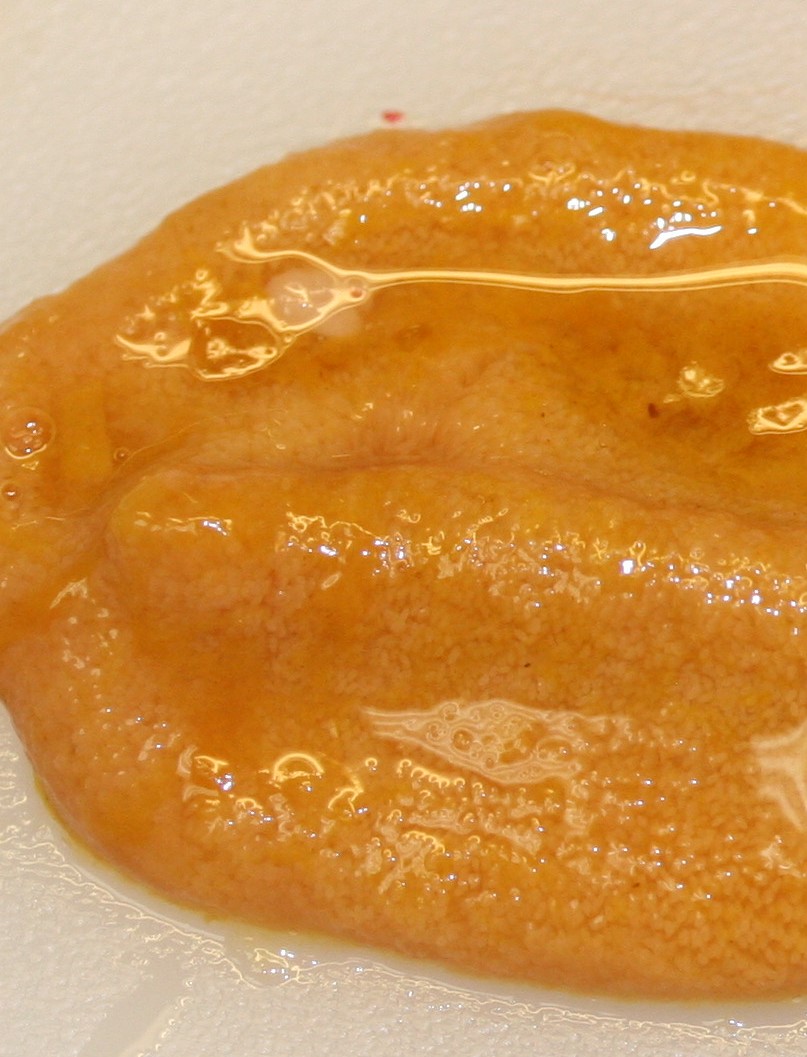

Supplement: Supplementary file 1 — Additional file 1. Additional photos of intestinal lesions. Folder with 47 additional photos in JPG format illustrating turkeys with intestinal lesions that were assigned to scores 0, 1, 2 or 3. [file 12917_2020_2270_MOESM1_ESM.zip › Score2 IdV7 Exp4 Duodenum.jpg]

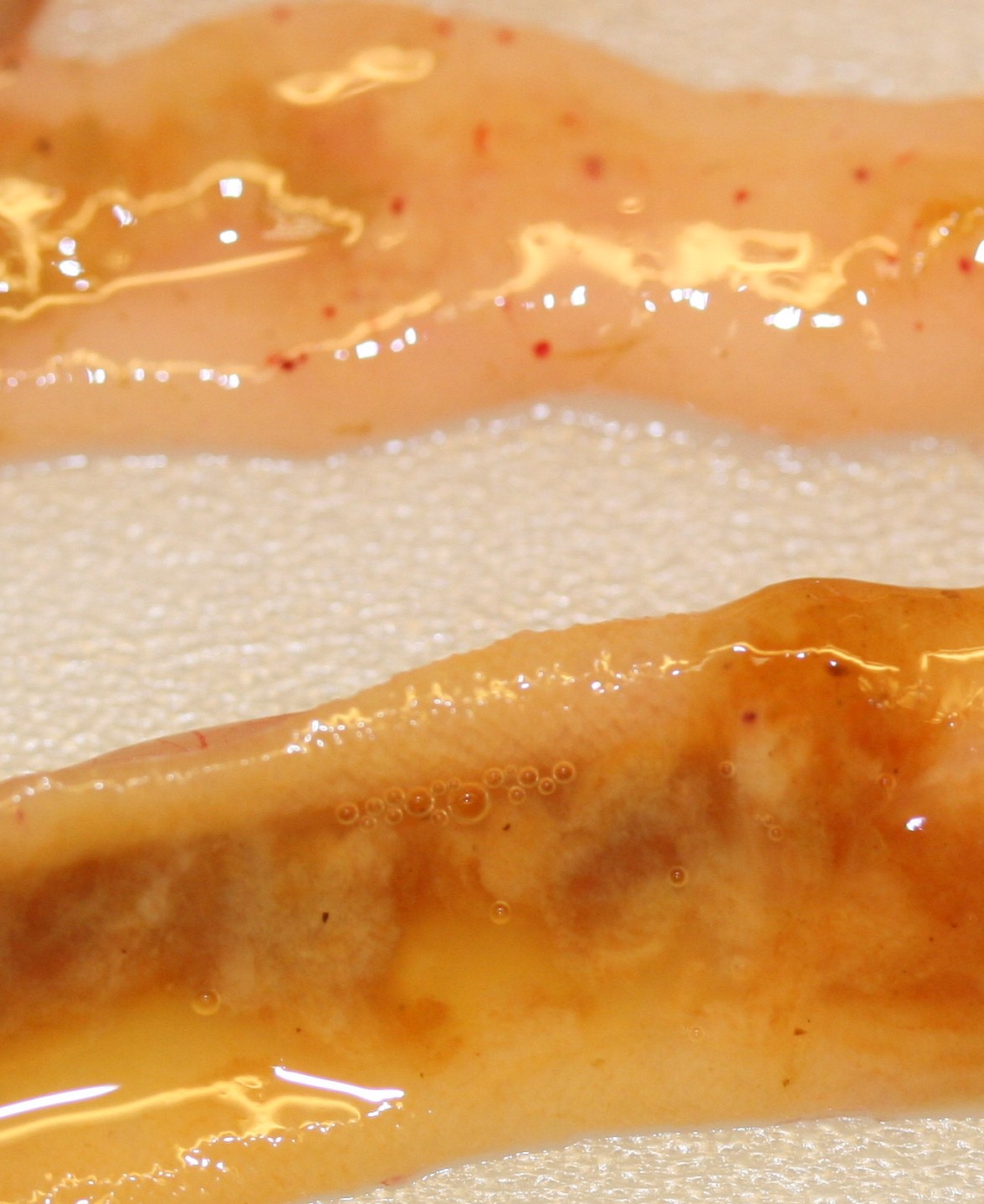

Supplement: Supplementary file 1 — Additional file 1. Additional photos of intestinal lesions. Folder with 47 additional photos in JPG format illustrating turkeys with intestinal lesions that were assigned to scores 0, 1, 2 or 3. [file 12917_2020_2270_MOESM1_ESM.zip › Score2 IdV7b Exp4.jpg]

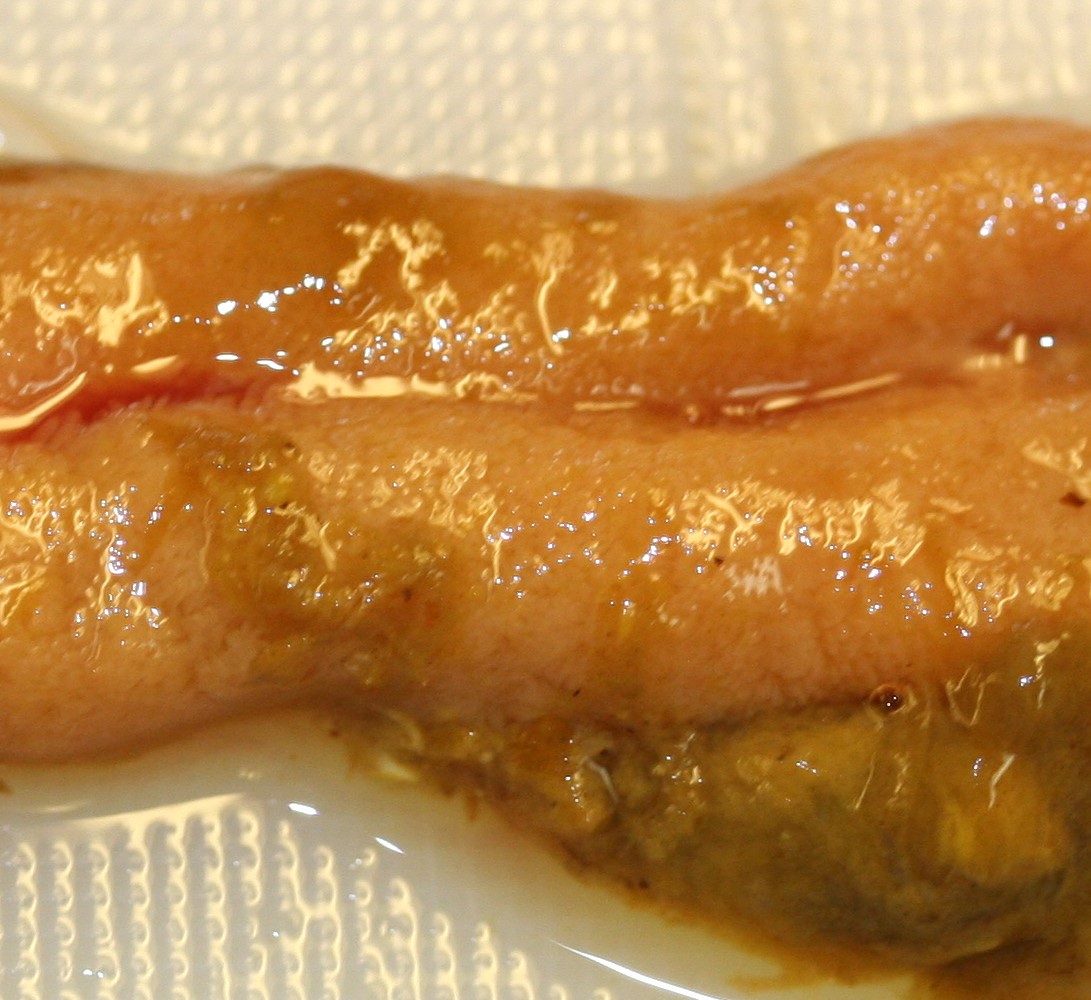

Supplement: Supplementary file 1 — Additional file 1. Additional photos of intestinal lesions. Folder with 47 additional photos in JPG format illustrating turkeys with intestinal lesions that were assigned to scores 0, 1, 2 or 3. [file 12917_2020_2270_MOESM1_ESM.zip › Score2 IdV8 Exp4.jpg]

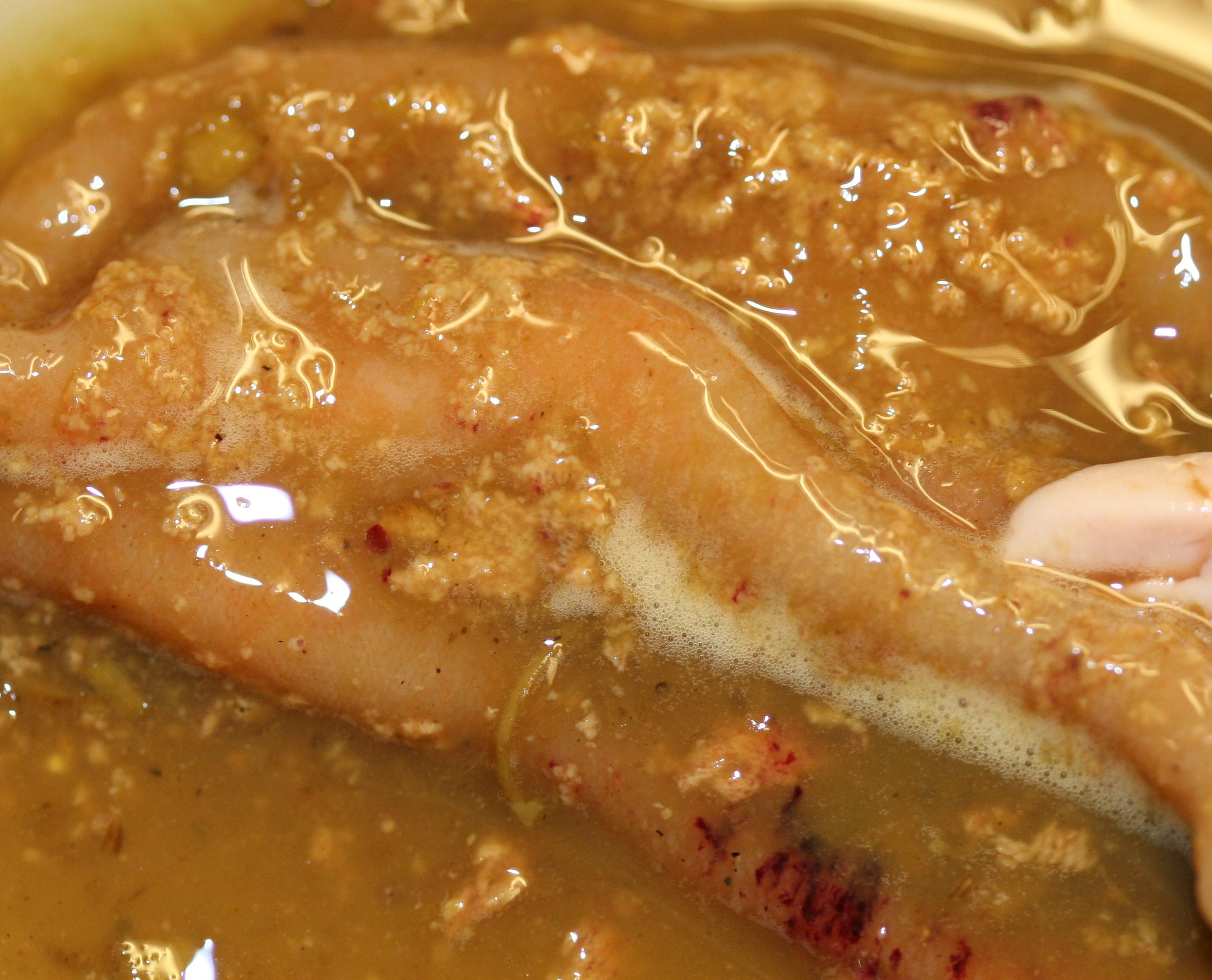

Supplement: Supplementary file 1 — Additional file 1. Additional photos of intestinal lesions. Folder with 47 additional photos in JPG format illustrating turkeys with intestinal lesions that were assigned to scores 0, 1, 2 or 3. [file 12917_2020_2270_MOESM1_ESM.zip › Score3 Id1 Exp1 .jpg]

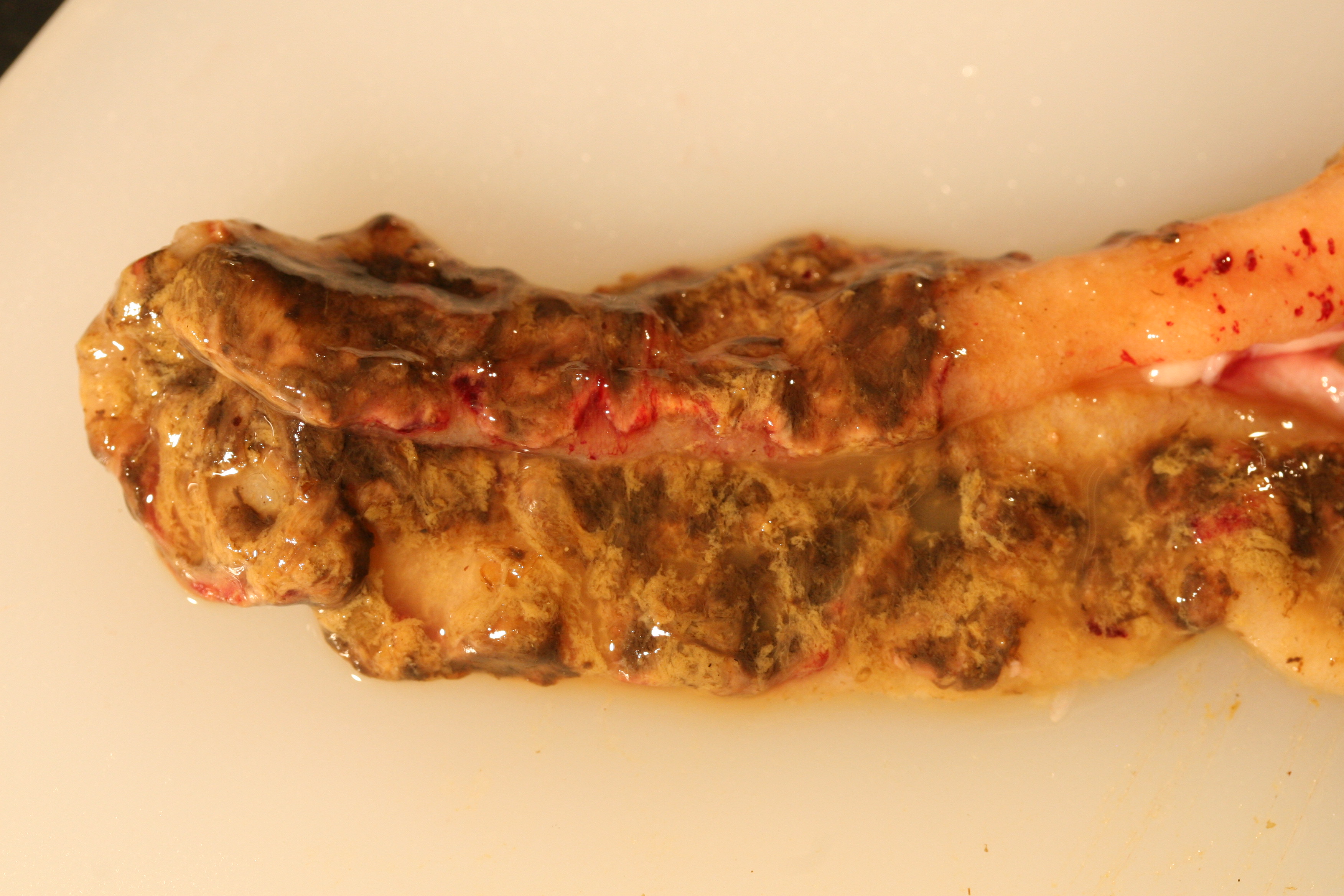

Supplement: Supplementary file 1 — Additional file 1. Additional photos of intestinal lesions. Folder with 47 additional photos in JPG format illustrating turkeys with intestinal lesions that were assigned to scores 0, 1, 2 or 3. [file 12917_2020_2270_MOESM1_ESM.zip › Score3 Id11 .jpg]

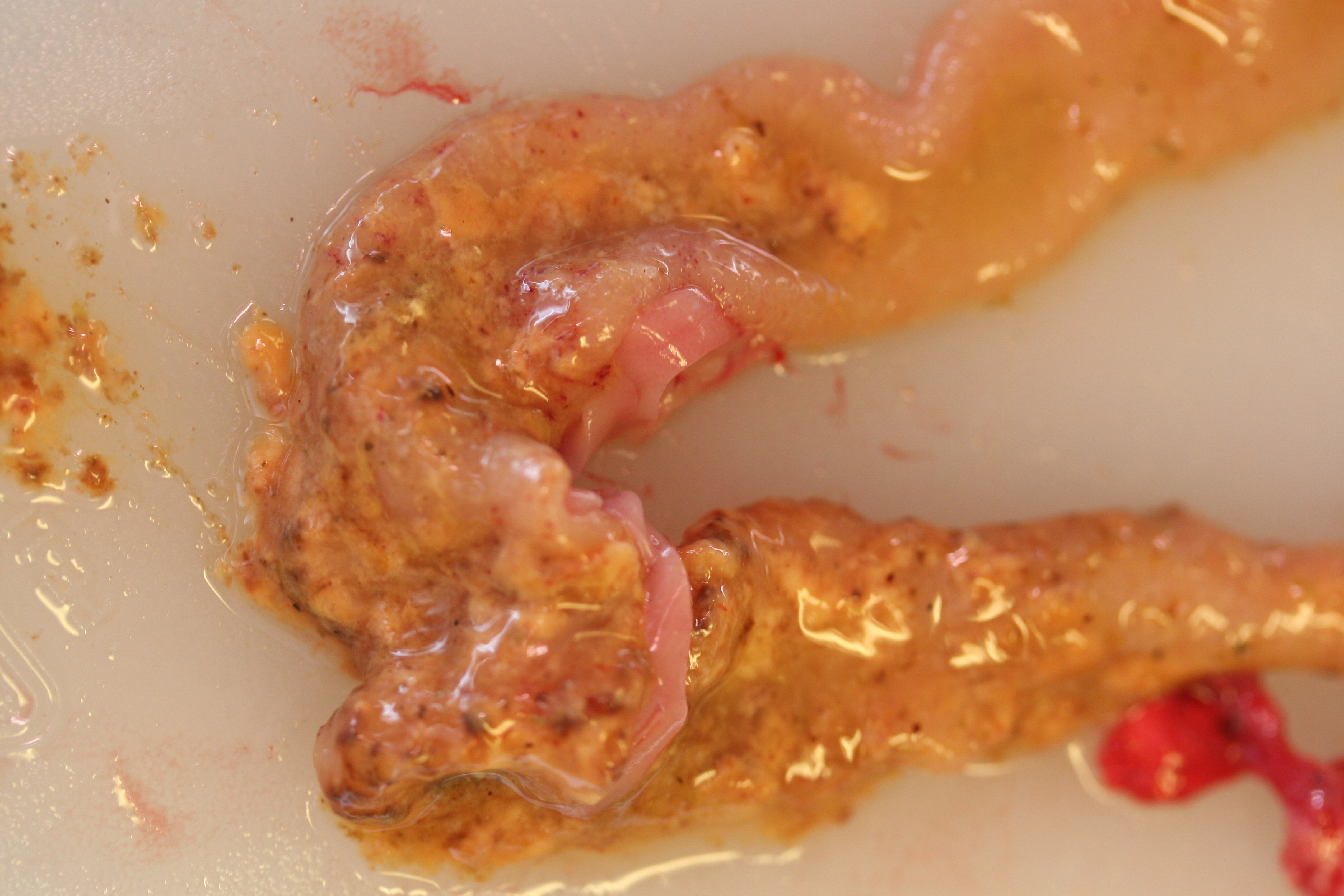

Supplement: Supplementary file 1 — Additional file 1. Additional photos of intestinal lesions. Folder with 47 additional photos in JPG format illustrating turkeys with intestinal lesions that were assigned to scores 0, 1, 2 or 3. [file 12917_2020_2270_MOESM1_ESM.zip › Score3 Id14 Exp2 .jpg]

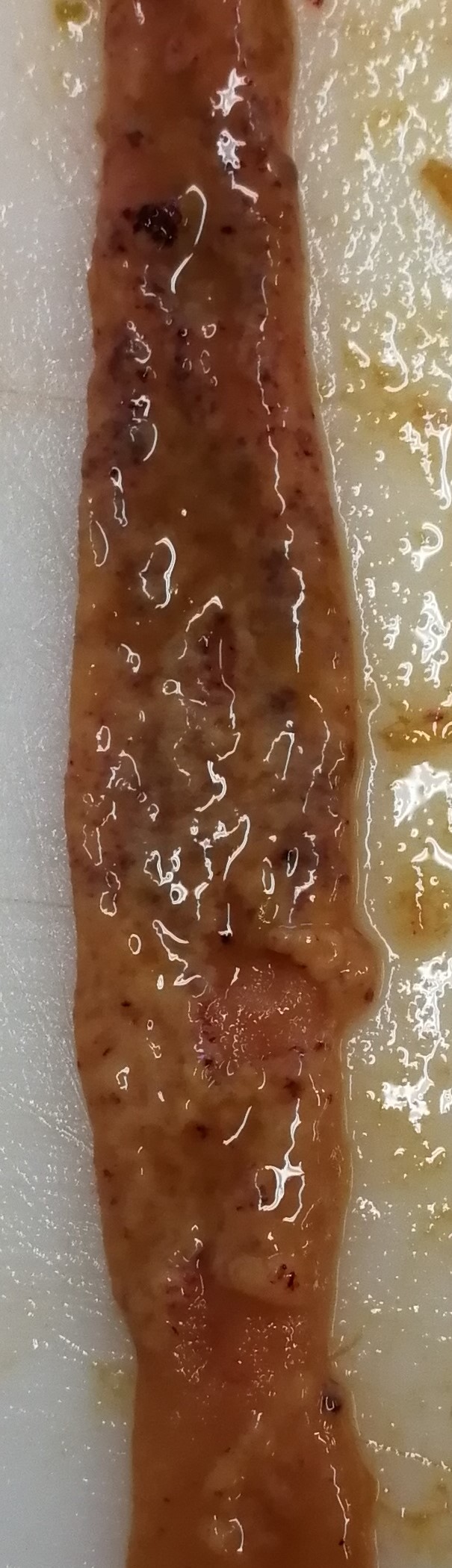

Supplement: Supplementary file 1 — Additional file 1. Additional photos of intestinal lesions. Folder with 47 additional photos in JPG format illustrating turkeys with intestinal lesions that were assigned to scores 0, 1, 2 or 3. [file 12917_2020_2270_MOESM1_ESM.zip › Score3 Id26 Exp9.jpg]

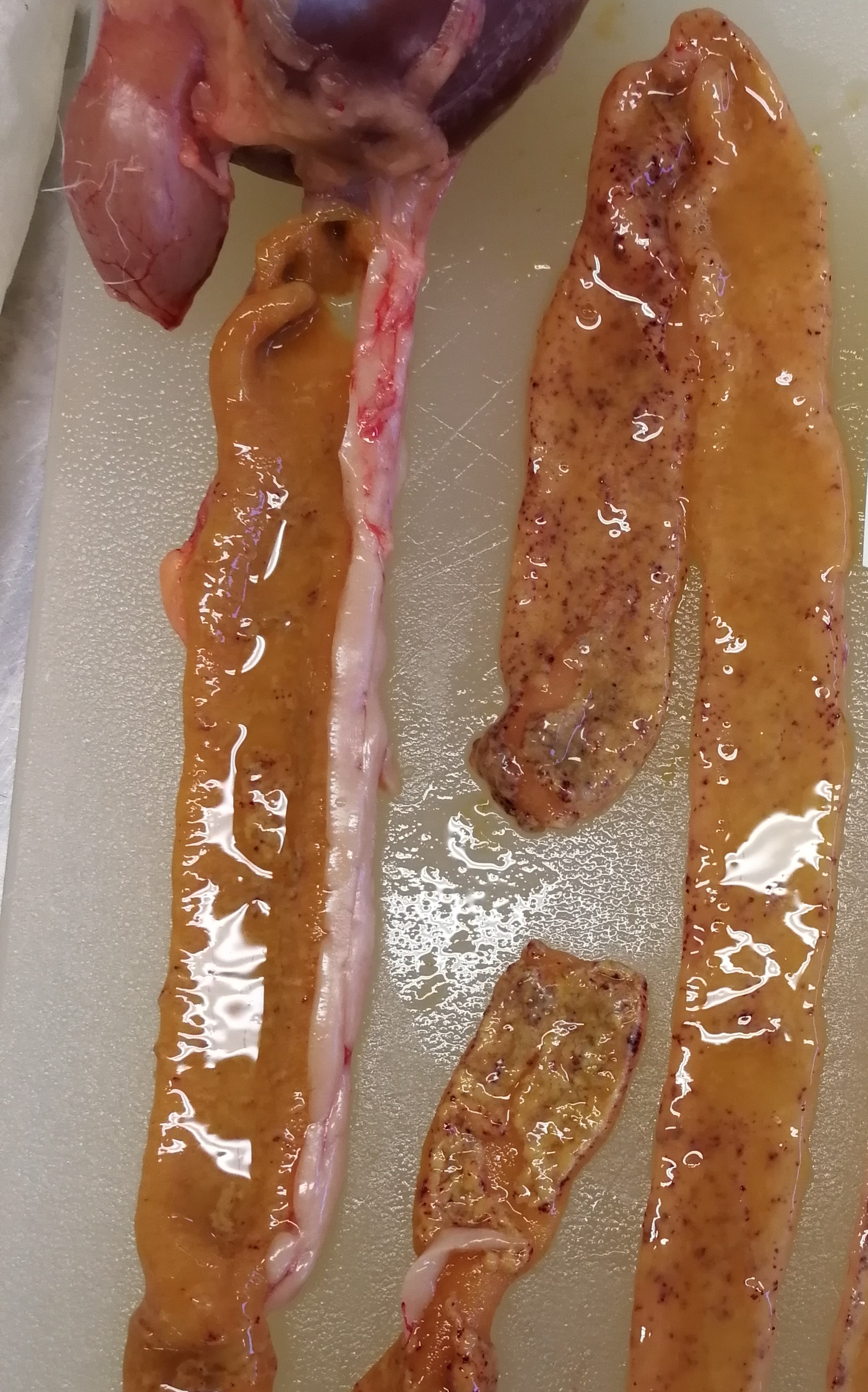

Supplement: Supplementary file 1 — Additional file 1. Additional photos of intestinal lesions. Folder with 47 additional photos in JPG format illustrating turkeys with intestinal lesions that were assigned to scores 0, 1, 2 or 3. [file 12917_2020_2270_MOESM1_ESM.zip › Score3 Id29 Exp9.jpg]

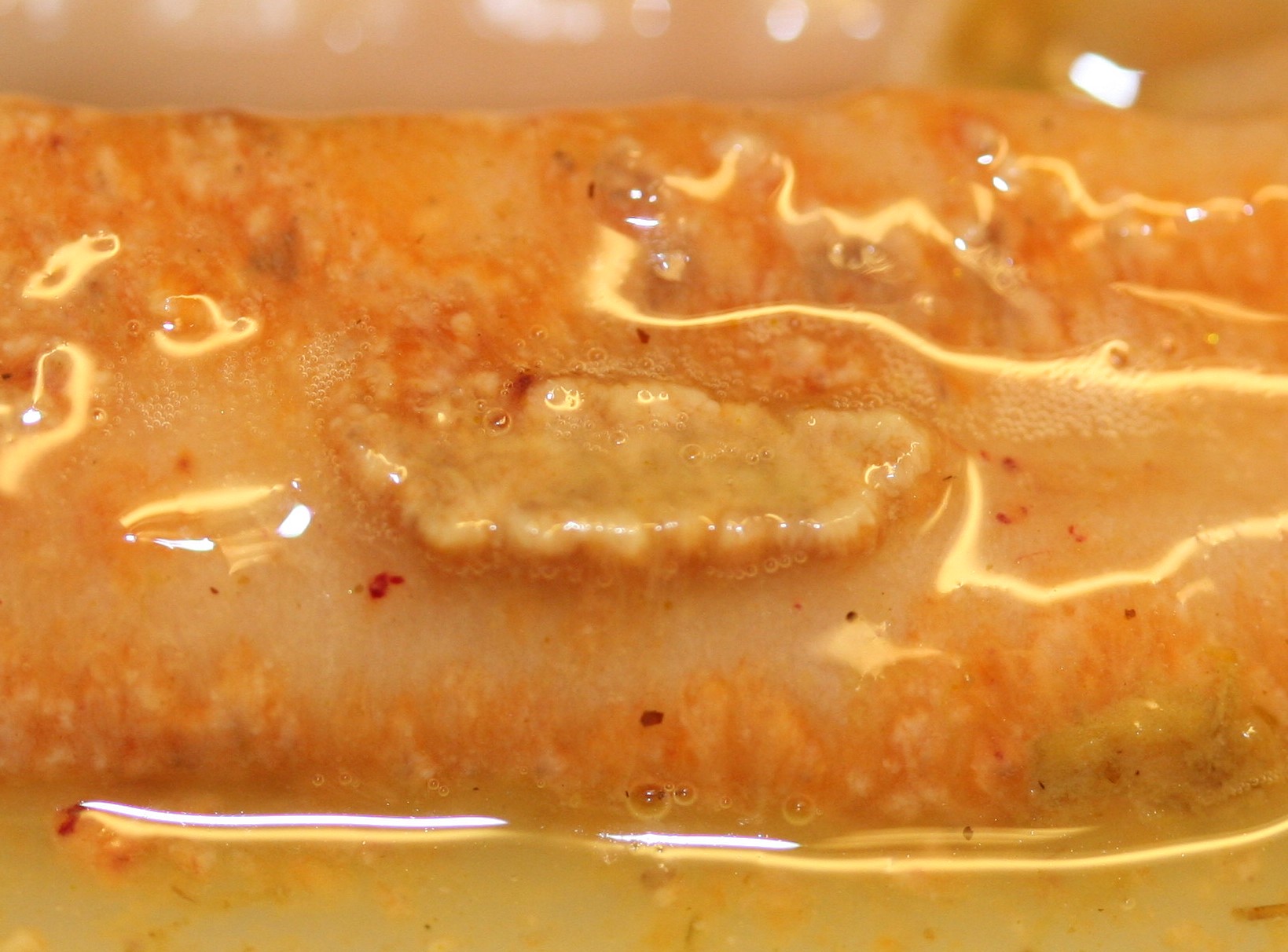

Supplement: Supplementary file 1 — Additional file 1. Additional photos of intestinal lesions. Folder with 47 additional photos in JPG format illustrating turkeys with intestinal lesions that were assigned to scores 0, 1, 2 or 3. [file 12917_2020_2270_MOESM1_ESM.zip › Score3 Id4 .jpg]

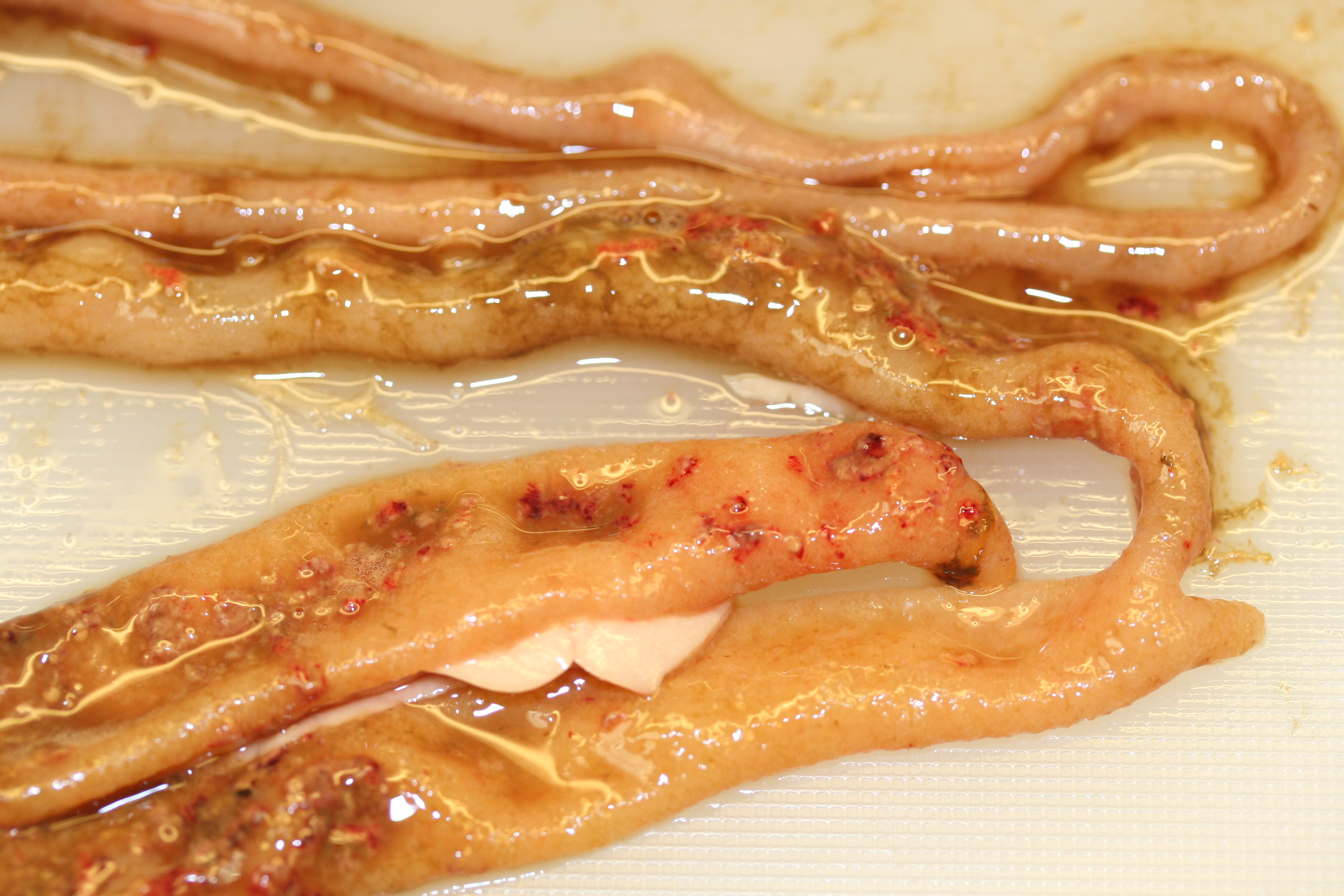

Supplement: Supplementary file 1 — Additional file 1. Additional photos of intestinal lesions. Folder with 47 additional photos in JPG format illustrating turkeys with intestinal lesions that were assigned to scores 0, 1, 2 or 3. [file 12917_2020_2270_MOESM1_ESM.zip › Score3 Id5 Exp4.jpg]

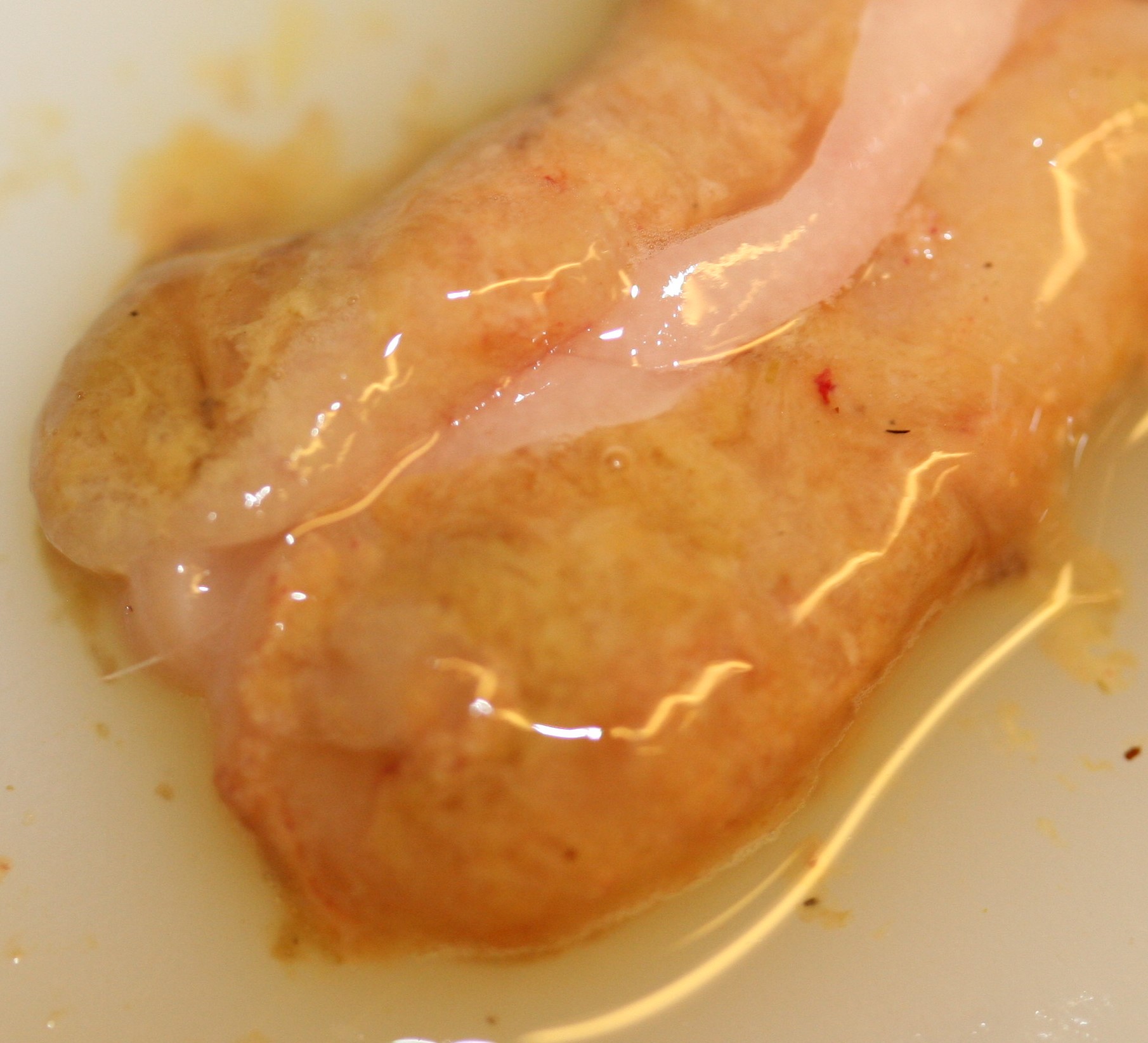

Supplement: Supplementary file 1 — Additional file 1. Additional photos of intestinal lesions. Folder with 47 additional photos in JPG format illustrating turkeys with intestinal lesions that were assigned to scores 0, 1, 2 or 3. [file 12917_2020_2270_MOESM1_ESM.zip › Score3 Id6 Exp1 .jpg]

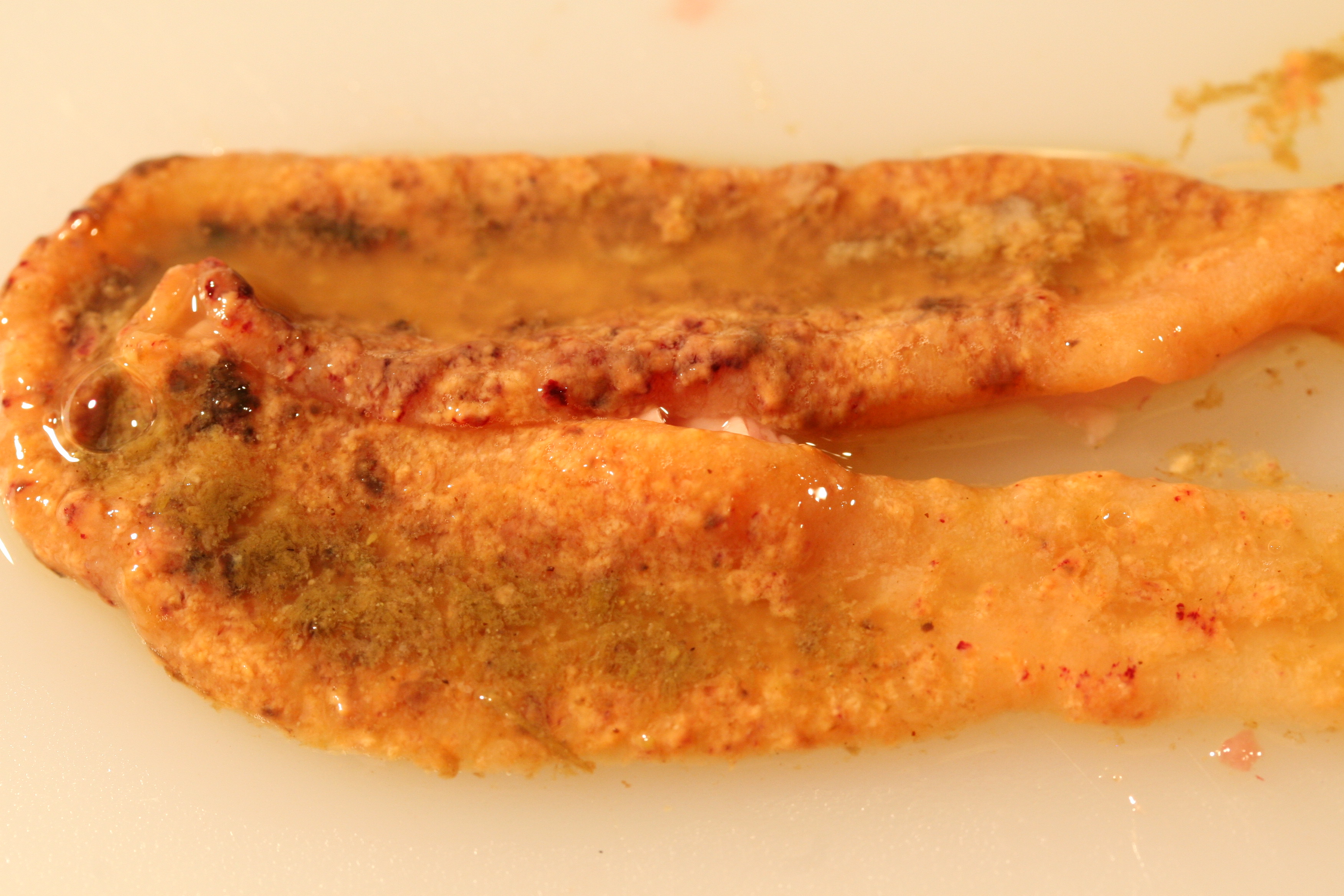

Supplement: Supplementary file 1 — Additional file 1. Additional photos of intestinal lesions. Folder with 47 additional photos in JPG format illustrating turkeys with intestinal lesions that were assigned to scores 0, 1, 2 or 3. [file 12917_2020_2270_MOESM1_ESM.zip › Score3 Id7 Exp1 .jpg]

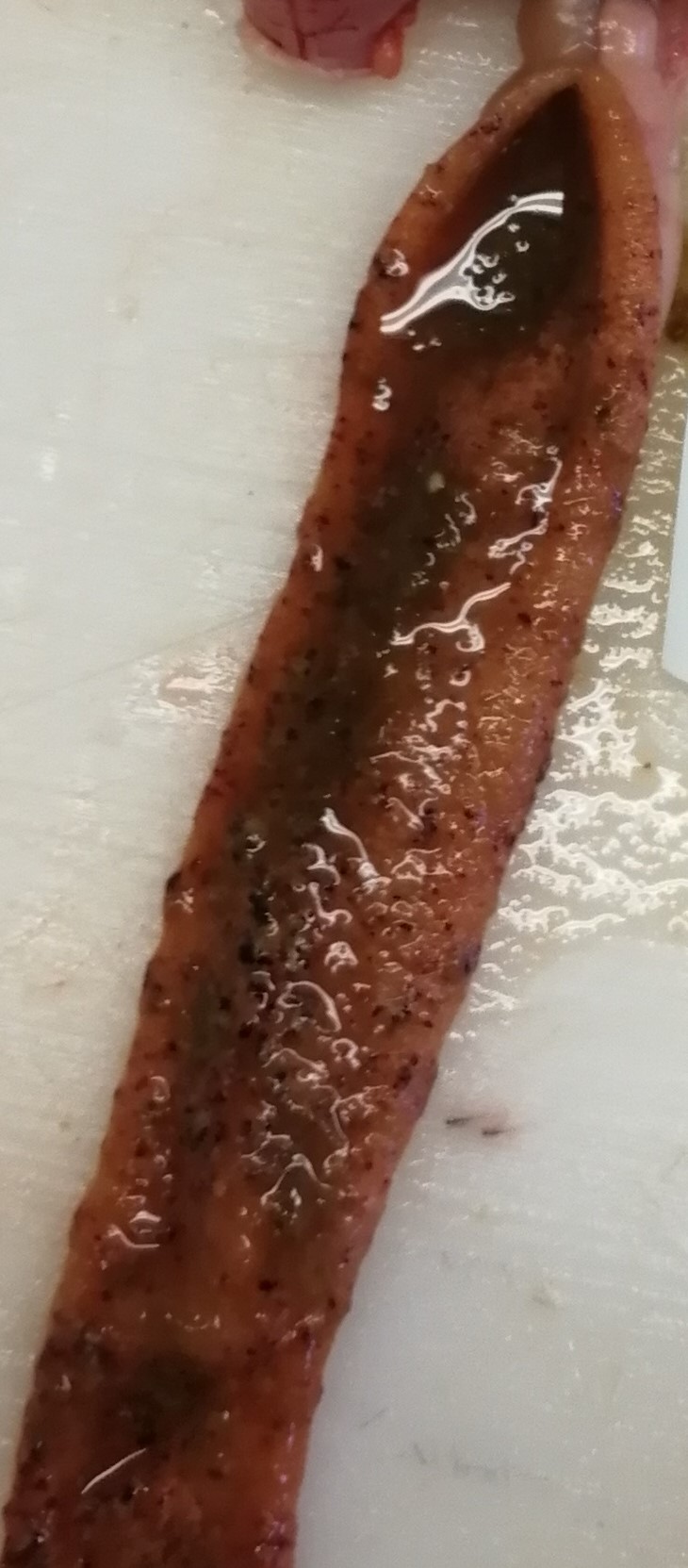

Supplement: Supplementary file 1 — Additional file 1. Additional photos of intestinal lesions. Folder with 47 additional photos in JPG format illustrating turkeys with intestinal lesions that were assigned to scores 0, 1, 2 or 3. [file 12917_2020_2270_MOESM1_ESM.zip › Score3 Id81a Exp9.jpg]

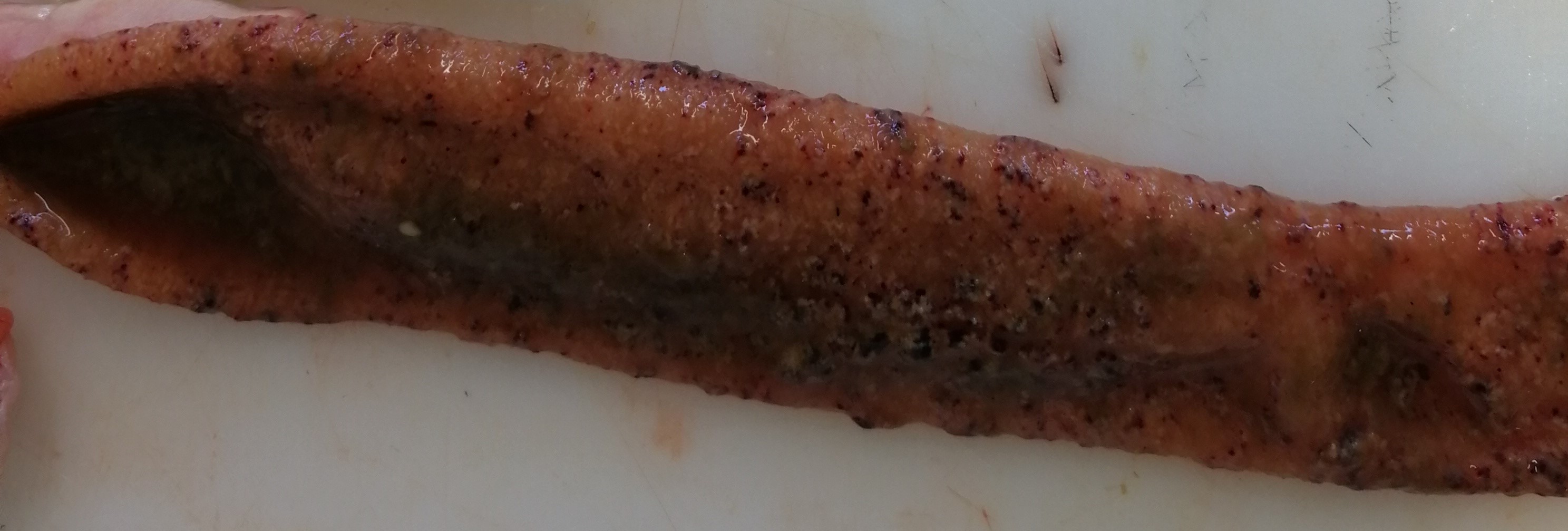

Supplement: Supplementary file 1 — Additional file 1. Additional photos of intestinal lesions. Folder with 47 additional photos in JPG format illustrating turkeys with intestinal lesions that were assigned to scores 0, 1, 2 or 3. [file 12917_2020_2270_MOESM1_ESM.zip › Score3 Id81c Exp9.jpg]

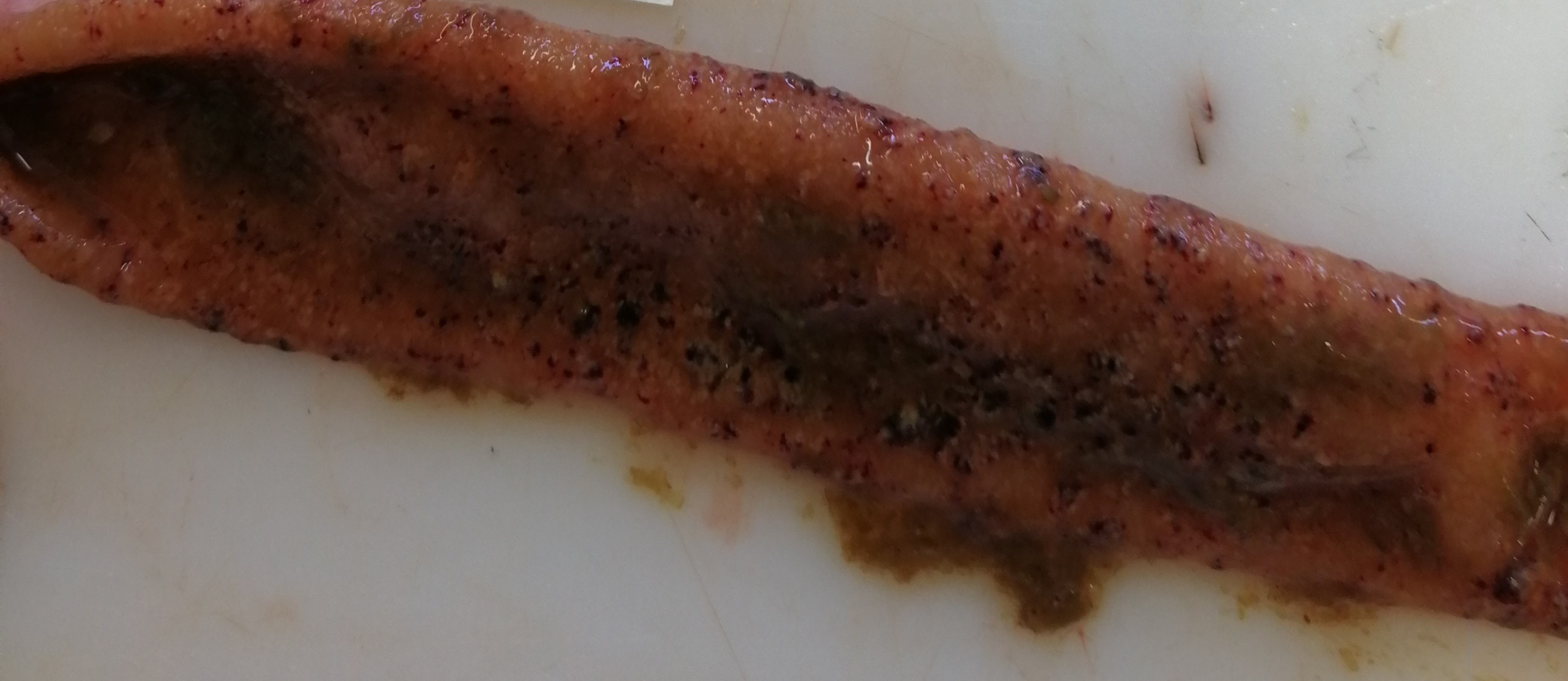

Supplement: Supplementary file 1 — Additional file 1. Additional photos of intestinal lesions. Folder with 47 additional photos in JPG format illustrating turkeys with intestinal lesions that were assigned to scores 0, 1, 2 or 3. [file 12917_2020_2270_MOESM1_ESM.zip › Score3 Id81d Exp9.jpg]

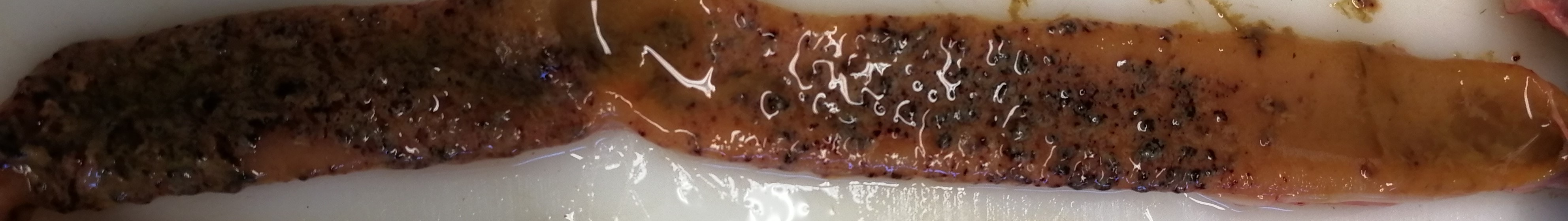

Supplement: Supplementary file 1 — Additional file 1. Additional photos of intestinal lesions. Folder with 47 additional photos in JPG format illustrating turkeys with intestinal lesions that were assigned to scores 0, 1, 2 or 3. [file 12917_2020_2270_MOESM1_ESM.zip › Score3 Id88 c Exp9.jpg]

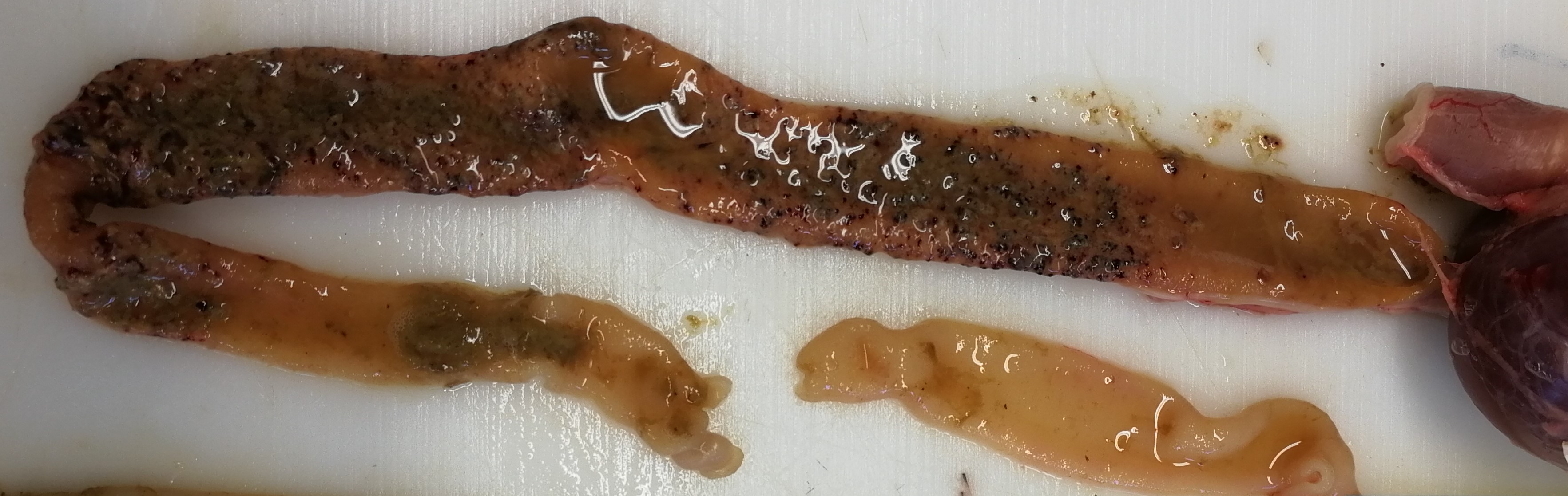

Supplement: Supplementary file 1 — Additional file 1. Additional photos of intestinal lesions. Folder with 47 additional photos in JPG format illustrating turkeys with intestinal lesions that were assigned to scores 0, 1, 2 or 3. [file 12917_2020_2270_MOESM1_ESM.zip › Score3 Id88a Exp9.jpg]

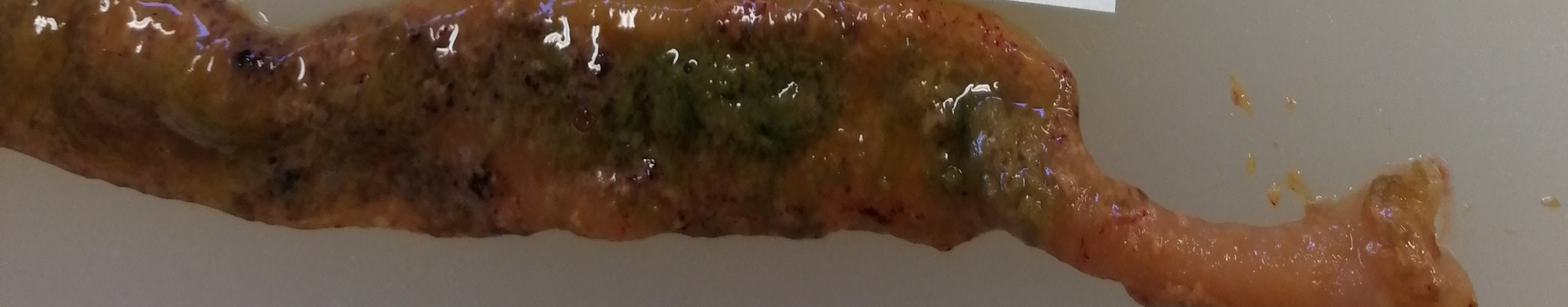

Supplement: Supplementary file 1 — Additional file 1. Additional photos of intestinal lesions. Folder with 47 additional photos in JPG format illustrating turkeys with intestinal lesions that were assigned to scores 0, 1, 2 or 3. [file 12917_2020_2270_MOESM1_ESM.zip › Score3 Id90b Exp9.jpg]

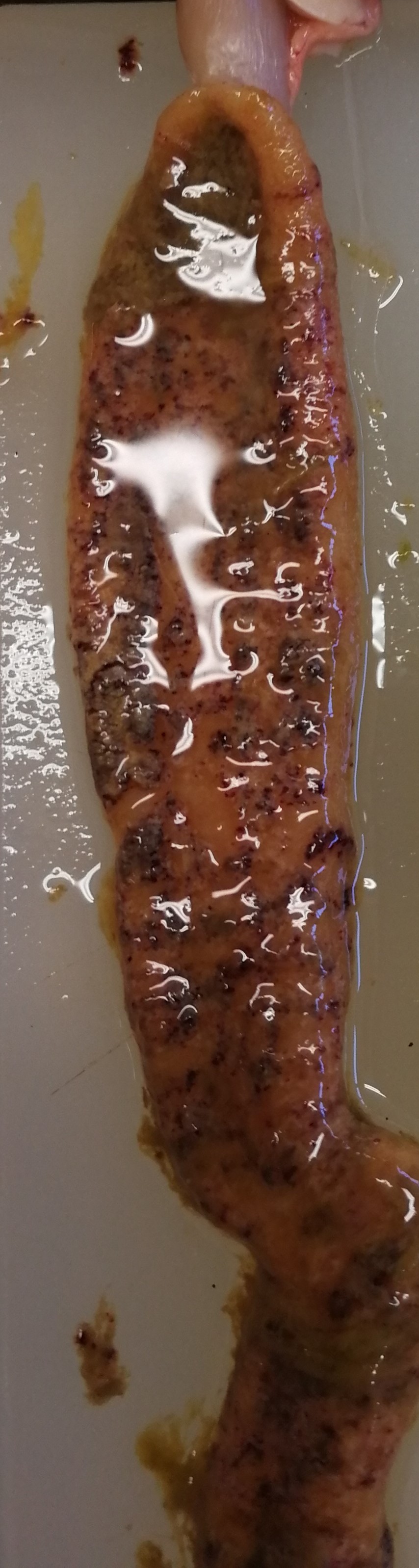

Supplement: Supplementary file 1 — Additional file 1. Additional photos of intestinal lesions. Folder with 47 additional photos in JPG format illustrating turkeys with intestinal lesions that were assigned to scores 0, 1, 2 or 3. [file 12917_2020_2270_MOESM1_ESM.zip › Score3 Id90c Exp9.jpg]

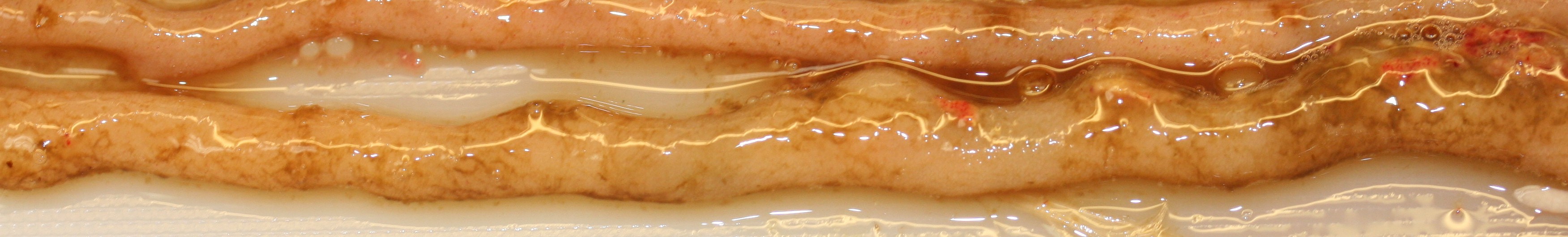

Supplement: Supplementary file 1 — Additional file 1. Additional photos of intestinal lesions. Folder with 47 additional photos in JPG format illustrating turkeys with intestinal lesions that were assigned to scores 0, 1, 2 or 3. [file 12917_2020_2270_MOESM1_ESM.zip › Score3 IdQ10e .jpg]

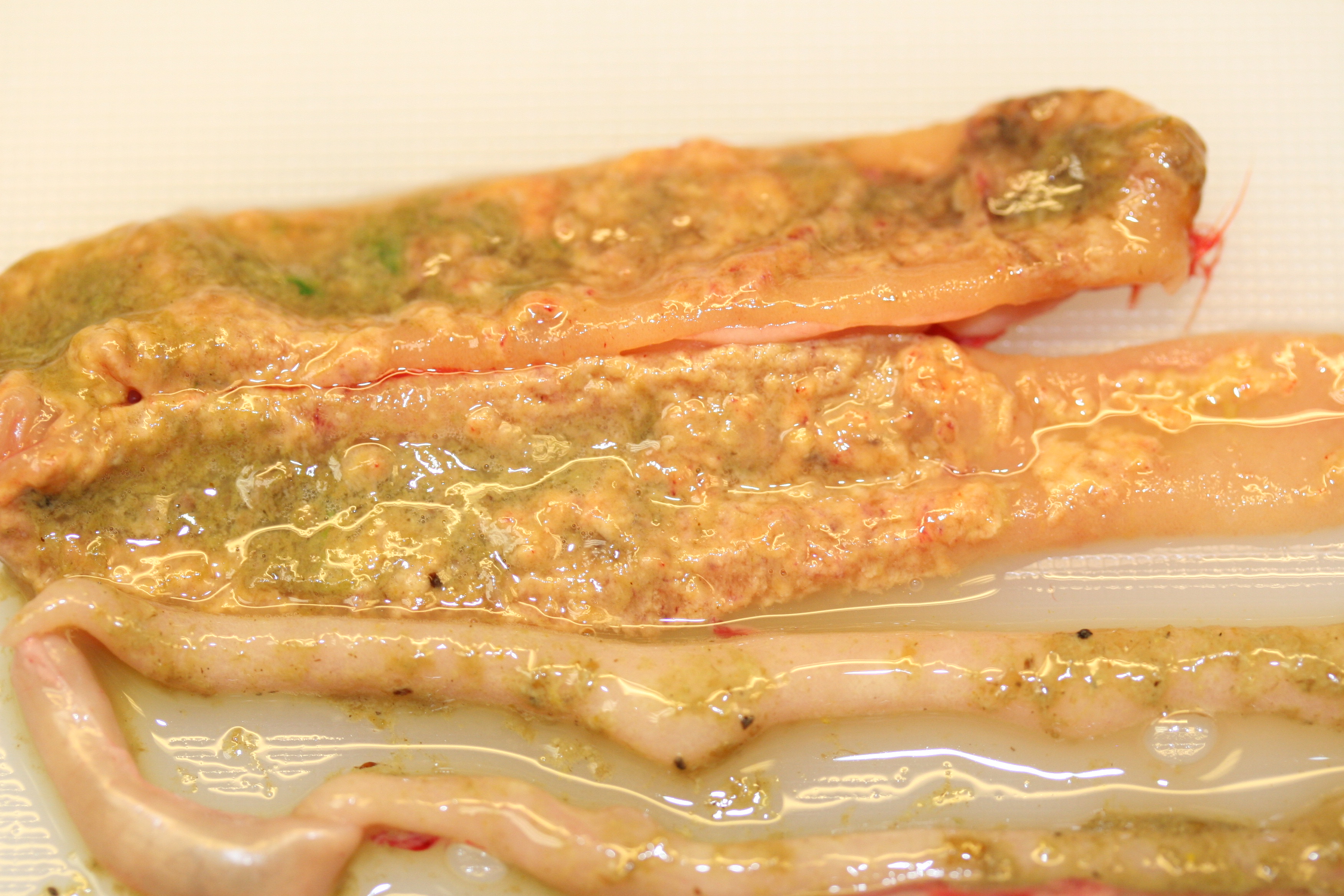

Supplement: Supplementary file 1 — Additional file 1. Additional photos of intestinal lesions. Folder with 47 additional photos in JPG format illustrating turkeys with intestinal lesions that were assigned to scores 0, 1, 2 or 3. [file 12917_2020_2270_MOESM1_ESM.zip › Score3 IdQ5d Exp4.jpg]

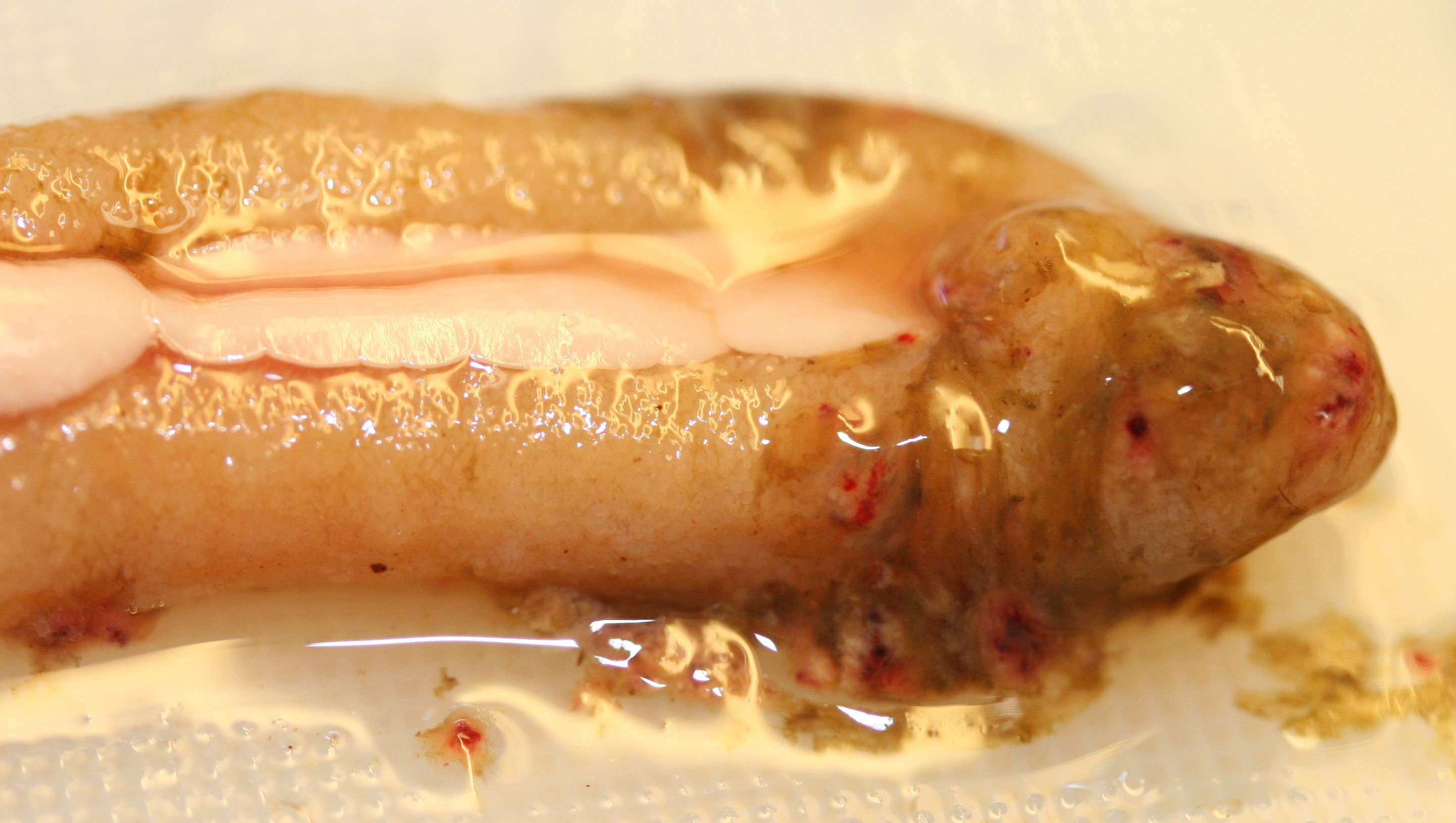

Supplement: Supplementary file 1 — Additional file 1. Additional photos of intestinal lesions. Folder with 47 additional photos in JPG format illustrating turkeys with intestinal lesions that were assigned to scores 0, 1, 2 or 3. [file 12917_2020_2270_MOESM1_ESM.zip › Score3 IdQ6g Exp4.jpg]

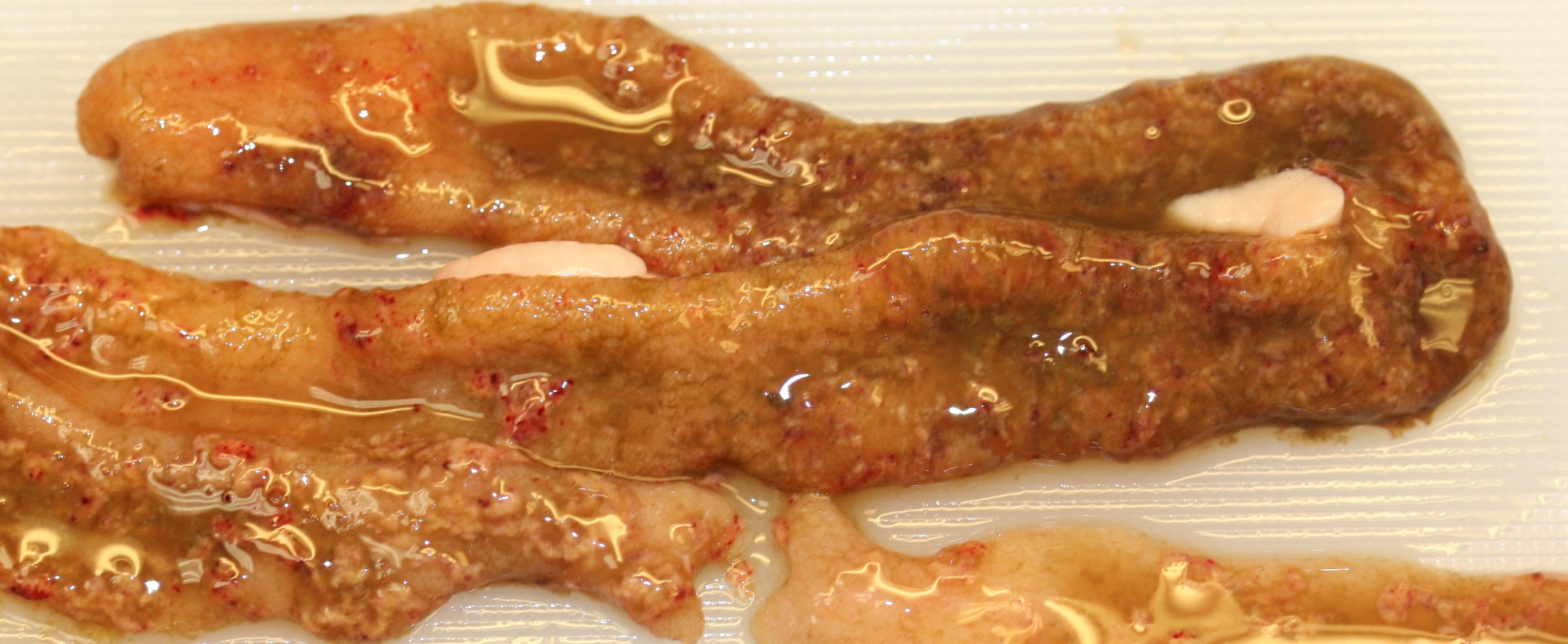

Supplement: Supplementary file 1 — Additional file 1. Additional photos of intestinal lesions. Folder with 47 additional photos in JPG format illustrating turkeys with intestinal lesions that were assigned to scores 0, 1, 2 or 3. [file 12917_2020_2270_MOESM1_ESM.zip › Score3 IdR5e Exp4 Duodenum.jpg]

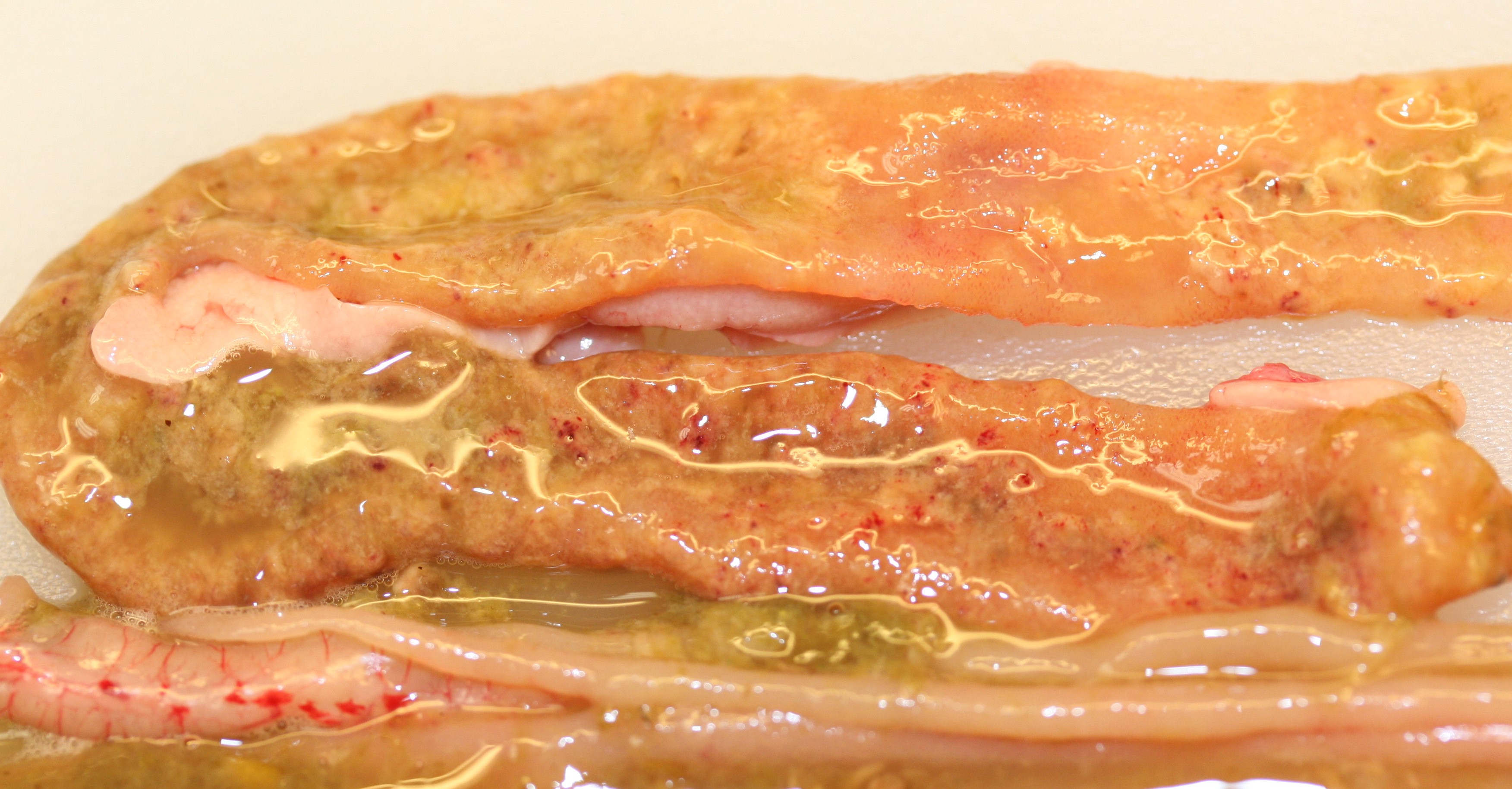

Supplement: Supplementary file 1 — Additional file 1. Additional photos of intestinal lesions. Folder with 47 additional photos in JPG format illustrating turkeys with intestinal lesions that were assigned to scores 0, 1, 2 or 3. [file 12917_2020_2270_MOESM1_ESM.zip › Score3 IdR7b Exp4.jpg]

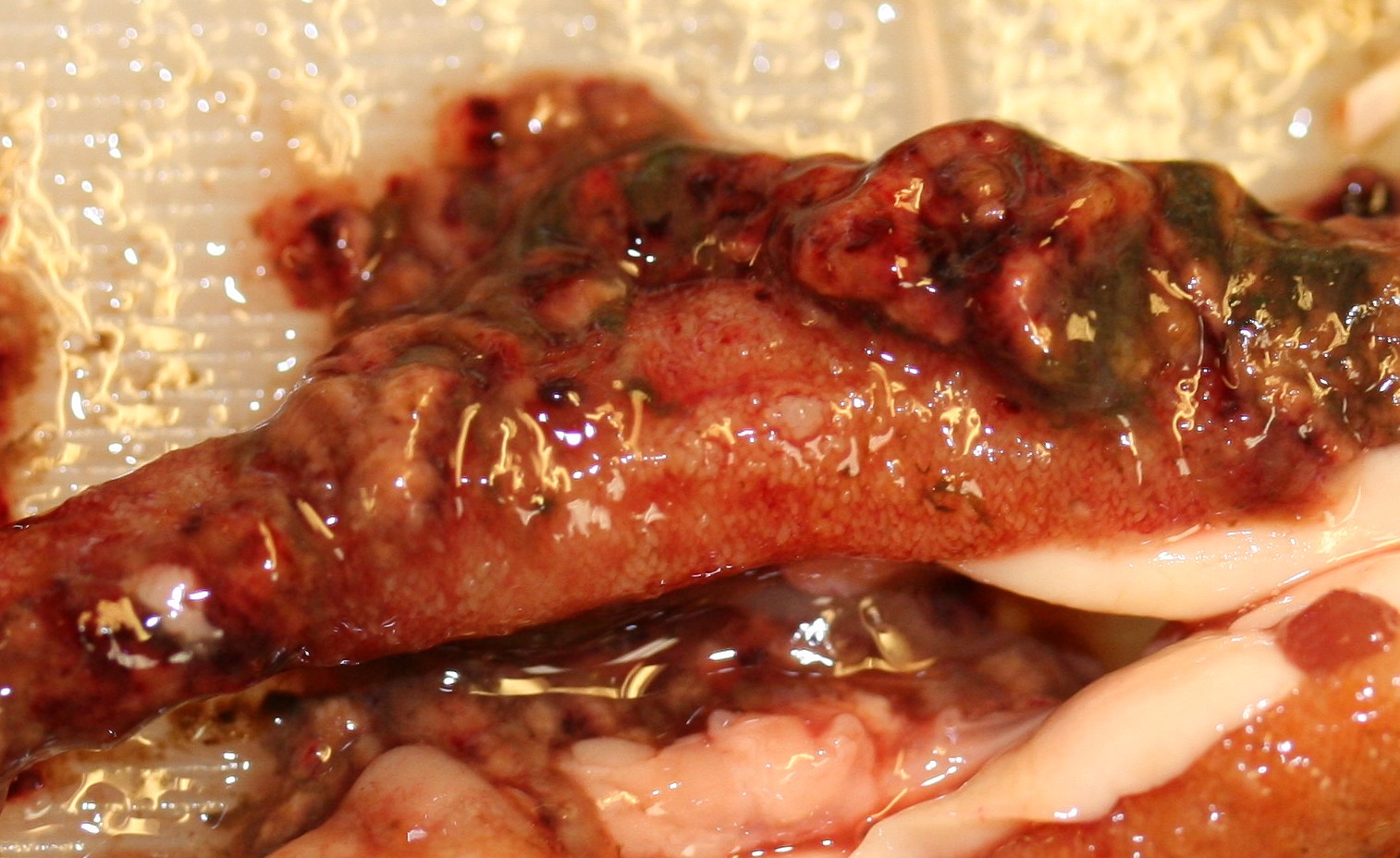

Supplement: Supplementary file 1 — Additional file 1. Additional photos of intestinal lesions. Folder with 47 additional photos in JPG format illustrating turkeys with intestinal lesions that were assigned to scores 0, 1, 2 or 3. [file 12917_2020_2270_MOESM1_ESM.zip › Score3 IdS11d Exp4.jpg]

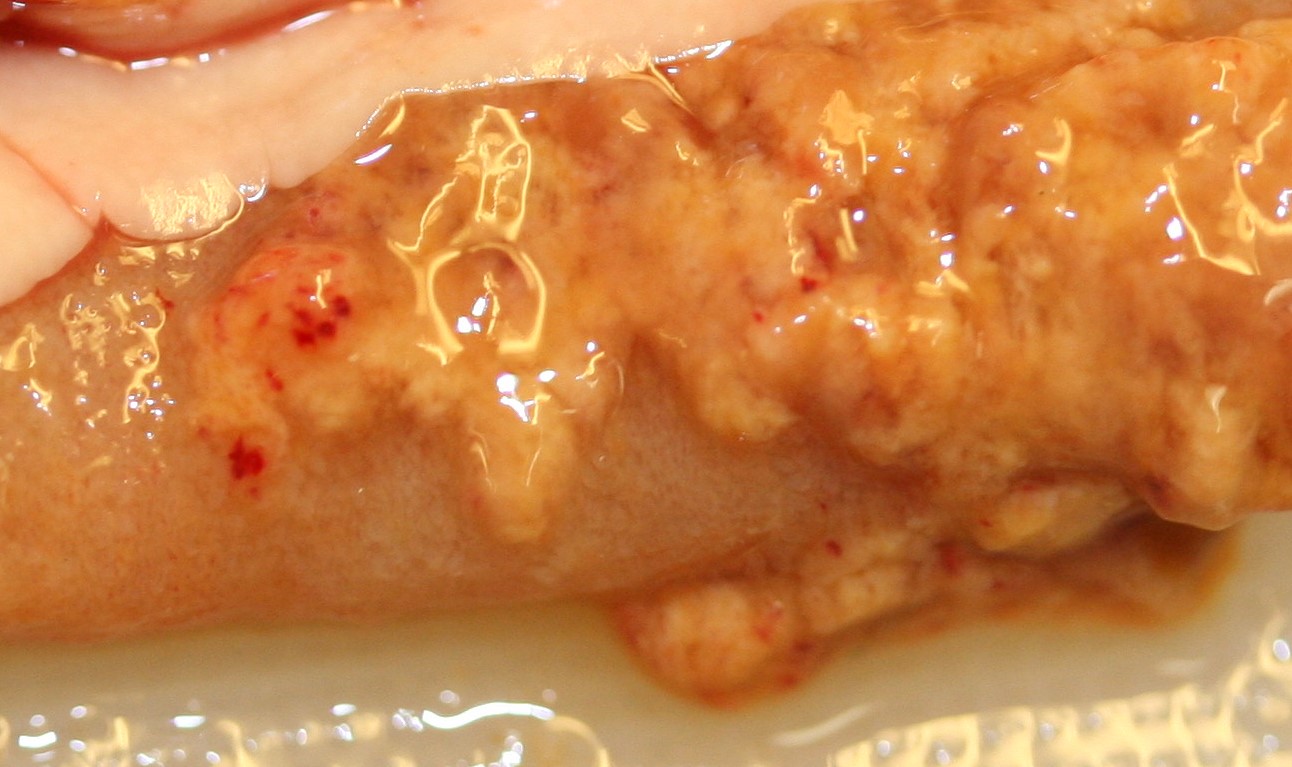

Supplement: Supplementary file 1 — Additional file 1. Additional photos of intestinal lesions. Folder with 47 additional photos in JPG format illustrating turkeys with intestinal lesions that were assigned to scores 0, 1, 2 or 3. [file 12917_2020_2270_MOESM1_ESM.zip › Score3 IdS5e Exp4 Duodenum.jpg]

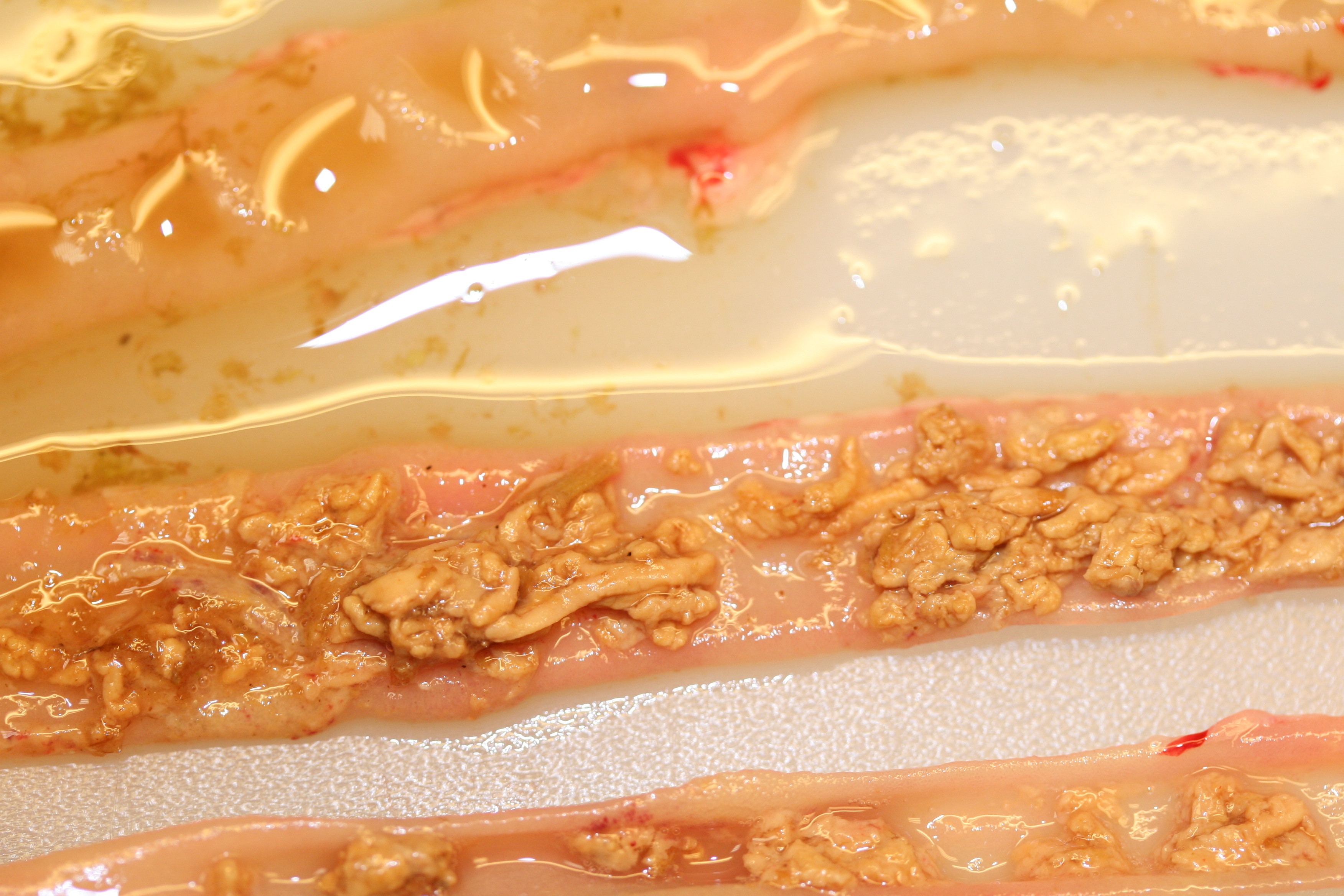

Supplement: Supplementary file 1 — Additional file 1. Additional photos of intestinal lesions. Folder with 47 additional photos in JPG format illustrating turkeys with intestinal lesions that were assigned to scores 0, 1, 2 or 3. [file 12917_2020_2270_MOESM1_ESM.zip › Score3 IdS8l Exp4.jpg]

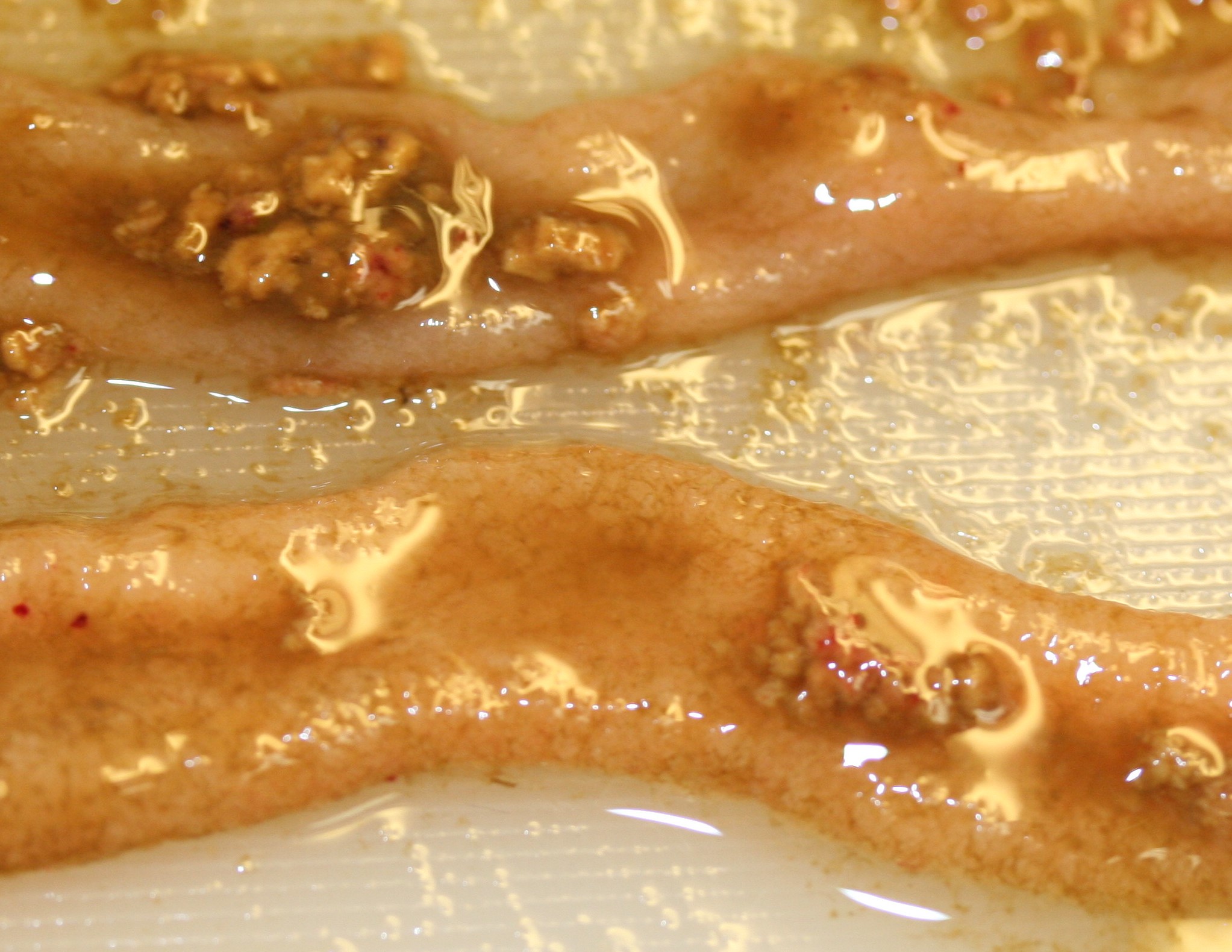

Supplement: Supplementary file 1 — Additional file 1. Additional photos of intestinal lesions. Folder with 47 additional photos in JPG format illustrating turkeys with intestinal lesions that were assigned to scores 0, 1, 2 or 3. [file 12917_2020_2270_MOESM1_ESM.zip › Score3 IdS9e Exp4 .jpg]

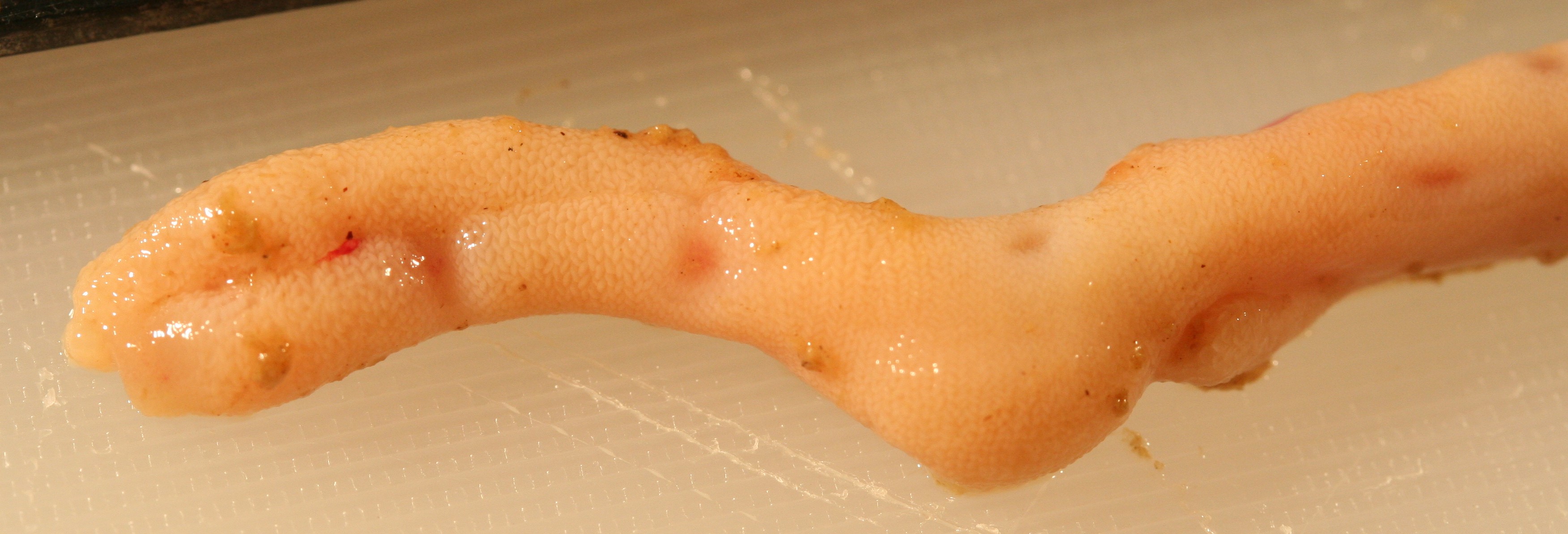

Supplement: Supplementary file 1 — Additional file 1. Additional photos of intestinal lesions. Folder with 47 additional photos in JPG format illustrating turkeys with intestinal lesions that were assigned to scores 0, 1, 2 or 3. [file 12917_2020_2270_MOESM1_ESM.zip › Score3 mucosal depressions Id61C IMG_3179 P704 .jpg]

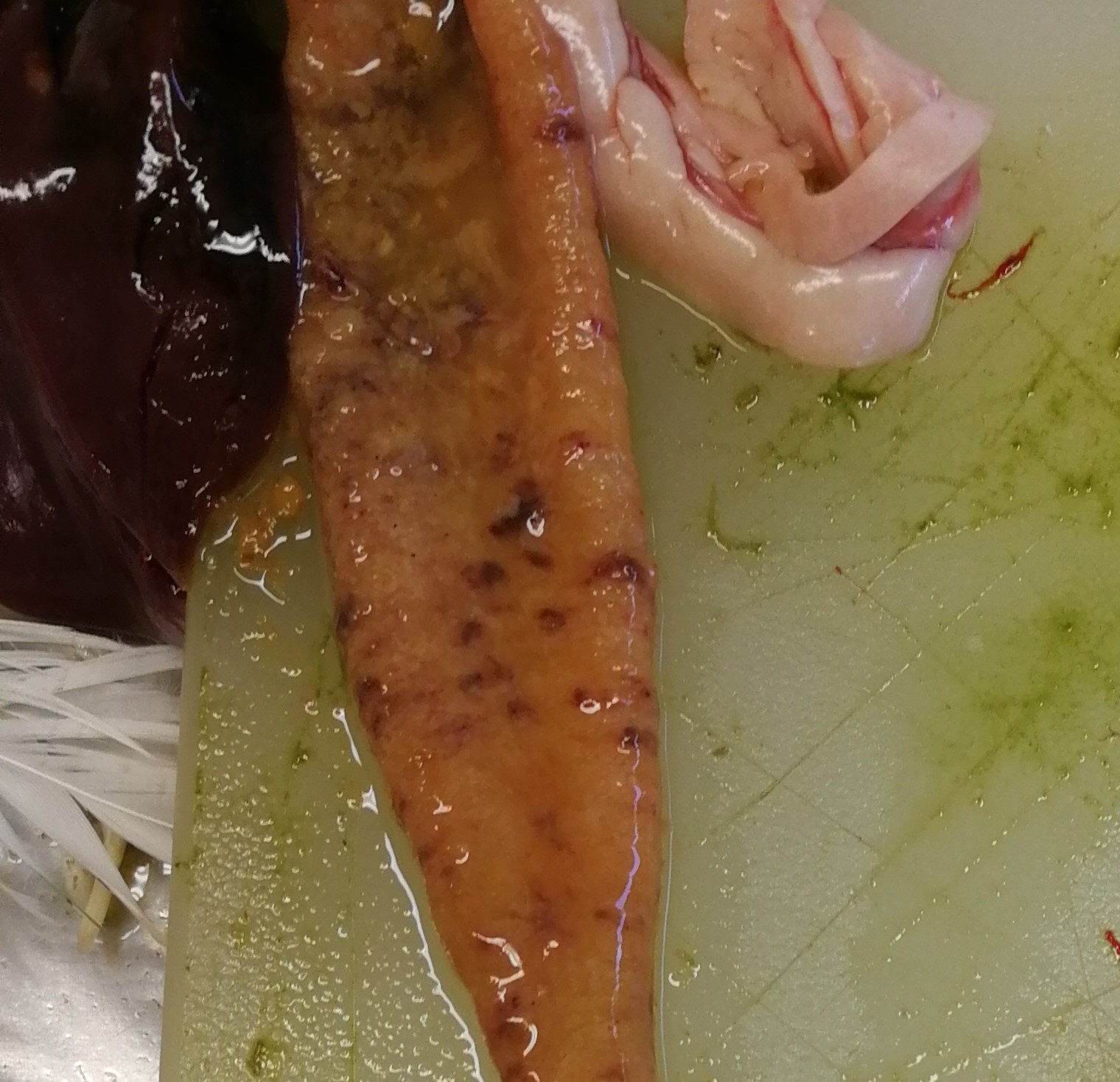

Supplement: Supplementary file 1 — Additional file 1. Additional photos of intestinal lesions. Folder with 47 additional photos in JPG format illustrating turkeys with intestinal lesions that were assigned to scores 0, 1, 2 or 3. [file 12917_2020_2270_MOESM1_ESM.zip › Score3 mucosal ulcers Id80 Exp9.jpg]
